# Supplementary material for: Integration of a multicomponent intervention for hypertension into primary healthcare services in Singapore—A cluster randomized controlled trial
Source: PLoS Med. 2022 Jun 13;19(6):e1004026. doi: 10.1371/journal.pmed.1004026 (PMC9239484; doi:10.1371/journal.pmed.1004026)
Supplement: S1 Appendix — Section S1: Research Contributions. Section S2: Nurse telephone follow-up checklist. Section S3: Physician Management Checklist. Section S4: Summary of intervention training. Section S5: Antihypertensive treatment algorithm. Section S6: Statistical methods for sensitivity for systolic blood pressure (BP). Section S7: Program delivery cost estimation—methods, assumptions, and data sources. Section S8: Tables A to G. Section S9. References. (DOCX) [file pmed.1004026.s002.docx]

**Supplementary Appendix**

Supplement to: Jafar TH, Tan NC, Shirore RM, et al. “Integration of a Multicomponent Intervention for Hypertension into Primary Healthcare Services in Singapore - A Cluster Randomised Controlled Trial”.

**S1 Protocol:**

Jafar TH, Tan NC, Allen JC, Finkelstein EA, Goh P, Moey P, et al. Management of hypertension and multiple risk factors to enhance cardiovascular health in Singapore: The SingHypertension cluster randomized trial. *Trials.* 2018;19(1):180. Epub 2018/03/16. doi: 10.1186/s13063-018-2559-x. PubMed PMID: 29540213;

<https://trialsjournal.biomedcentral.com/articles/10.1186/s13063-018-2559-x>

**S1 Statistical Analysis Plan:**

Allen JC, Jr., Halaand B, Shirore RM, Jafar TH, for SingHypertension Study G. Statistical analysis plan for management of hypertension and multiple risk factors to enhance cardiovascular health in Singapore: the SingHypertension pragmatic cluster randomized controlled trial. *Trials.* 2021;22(1):66. Epub 2021/01/21. doi: 10.1186/s13063-020-05016-4. PubMed PMID: 33468225.

<https://trialsjournal.biomedcentral.com/articles/10.1186/s13063-020-05016-4>

Table of Contents

[Section S1: Research Contributions 3](#_Toc104466072)

[Section S2: Nurse telephone follow-up checklist 5](#_Toc104466073)

[Section S3: Physician management checklist 9](#_Toc104466074)

[Section S4: Summary of intervention training 11](#_Toc104466075)

[Section S5: Antihypertensive treatment algorithm ^1,2,3,4^ 12](#_Toc104466076)

[Section S6: Statistical methods for sensitivity for systolic blood pressure (BP) 14](#_Toc104466077)

[Section S7: Program delivery cost estimation – methods, assumptions, and data sources 16](#_Toc104466078)

[Section S8: Supplementary tables 22](#_Toc104466079)

[Table A. Baseline Characteristics 22](#_Toc104466080)

[Table B. Implementation and Intervention Fidelity 25](#_Toc104466081)

[Table C. Intervention Effect on Secondary Outcomes per ITT ^a^ 26](#_Toc104466082)

[Table D. Adverse and Serious Adverse Events by Intervention Group 29](#_Toc104466083)

[Table E. Intraclass correlation coefficient (ICC) of outcomes measured 30](#_Toc104466084)

[Table F. Raw means (SD) of primary and BP-Related Key Secondary Outcomes 31](#_Toc104466085)

[Table G. Program Delivery Costs in Singapore Dollar (SGD) 32](#_Toc104466086)

[Section S9. References: 34](#_Toc104466087)

# Section S1: Research Contributions

**Main PI and site PI:** Professor Tazeen H Jafar (overall principal investigator), Dr Tan Ngiap Chuan (main Co-PI).

**SingHypertension Study Group Members:**

1. **Duke-NUS Medical School (coordinating center):**

Professor Tazeen H Jafar (overall principal investigator, clinician-scientist/health systems trialist), Assistant Professor John Allen Jr (trial statistician), Professor Eric Finkelstein (health economist), Dr Rupesh Shirore (research associate, data manager and analyst), Dr. Chandrika Ramakrishnan (senior research associate and project manager), Dr Yeli Wang (research fellow), Ms. Cecille Lintag (administrative executive).

1. **SingHealth Polyclinics (SHP)**
   1. **SHP Office of Research:** Dr Tan Ngiap Chuan (SingHealth Polyclinics principal investigator, main Co-PI), Ms Patricia T Kin (research administration), Ms Tan Yang Thong Caris (research administration), Ms Reena Chandhini Subramanian (clinical research coordinator) Ms Chris Goh Wan Teng (clinical research coordinator), Ms Liu Jianying (clinical research coordinator), Ms Parames Khalechelvam (clinical research coordinator), Ms Usha Sankari (research administration), Ms Ashma Rafi Sayyad (clinical research coordinator).
   2. **SHP study sites**: Dr Moey Kirm Seng Peter (Site PI), Dr Hwang Siew Wai (Site PI), Dr Quah Hui Min Joanne (Site PI), Dr Goh Soo Chye Paul (Site PI), Dr Kanika Tyagi (Site PI), Dr Koong Ying Leng Agnes (Site PI), Dr Kang Chun-Yun Gary (Site PI), Dr Koot David (Site Co-I), Ms Anna Purani D/O Raman Singarasoo (Site Co-I), Dr Lee Cia Sin (Site Co-I), Ms Kala D/O Ramasamy (Site Co-I), Dr Hee Pei Ru Hilda (Site Co-I), Ms Chia Puay Kim (Site Co-I), Dr Wee Yi-Mei Sabrina (Site Co-I), Ms Zuhaida Binte Amir (Site Co-I), Dr Siti Sarina Binte Mohd Sairazi (Site Co-I), Ms Fazlina Binte Fesal (Site Co-I), Dr Yap Chee Mun (Site Co-I), Ms Vasanthi D/O Sokalingam (Site Co-I), Dr Chong Oi Fong (Site Co-I), Ms Fong Mew Keng (Site Co-I), Ms Xiang Ying (Site Co-I), Ms Bavani D/O Chandra (study team member), Ms Herwanny Binte Omar (study team member), Ms Ow Yong Wei Lin Jessica (study team member), Ms Tan Sok Heah (study team member), Mrs Suasini D/O Letchumanan (study team member), Mrs Tan Kwong Kent (study team member), Ms Irmawati Bte Ardzi (study team member), Ms Low Sok Yen Agnes (study team member), Ms Ng Swee Peng (study team member), Ms Tan Mui Kiang Sandy (study team member), Mrs Diana Binte Mohamed Yusoff (study team member), Ms Koh Ai Keng Lindy (study team member), Ms Tye Yee Mee (study team member), Ms Zhao Juan (study team member), Ms Elizabeth Ruth Chris (study team member), Ms Deng Shubin (study team member), Ms Ng Wan Bee (study team member), Ms Yap Sok Tin (study team member).
2. **National University Polyclinic** **study site**: Dr Anandan Gerard Thiagarajah (Site PI), Dr Santosh Lional Thomas (Site Co-I), Mrs Nirmala Arunkumar (Site Co-I).
3. **Shan You Counselling Centre (SYCC) Singapore:** Dr Cecilia Chu (Clinical psychologist).

**Source of funding**: National Medical Research Council (NMRC), Ministry of Health, Singapore.

**Data safety & monitoring board committee**: Dr. Tan Ru San (Chair), Professor Doris Young, Professor Vathsala Anantharaman, Dr. Edwin Chan Shih Yen.

# Section S2: Nurse telephone follow-up checklist

***Instructions:*** *At least 3 attempts should be made to make a telephone call in case of “No Answer” from the participant. Please fill the following information in case of lost-to follow-up:*

| **Attempt number** | **Date (dd/mm/yyyy)** | **Time (hh:mm)** |
| --- | --- | --- |
| 1 |  |  |
| 2 |  |  |
| 3 |  |  |

***Instructions:*** *If the telephone call is answered then introduce yourself and mention the name of the clinic from where you are calling. Remind the participant about the purpose of the call and time required to answer the questions. If agreed proceed to section A, else make an appointment for another call as per the participant’s convenience and enter details below:*

| **Appointment details** | |
| --- | --- |
| **Preferred date (dd/mm/yyyy)** | **Preferred time (hh:mm)** |
|  |  |

| **Section A: Details of telephone follow-up** | | |
| --- | --- | --- |
| **No.** | **Item** | **Response** |
| A1 | Name of the nurse clinician or nurse who conduct the telephone follow-up |  |
| A2 | Date (dd/mm/yyyy) | _ _ / _ _ / _ _ _ _ |
| A3 | Interview start time (hh:mm) |  |
| A4 | Interview end time (hh:mm) |  |
| A5 | Baseline BMI |  |
| A6 | Follow-up number | □ 1^st^ follow-up (after 1 month of enrolment)  □ 2^nd^ follow-up (after 2 months of enrolment)  □ 3^rd^ follow-up (after 3 months of enrolment)  □ 4^th^ follow-up (after 6 months of enrolment)  □ 5^th^ follow-up (after 9 months of enrolment)  □ 6^th^ follow-up (after 12 months of enrolment)  □ 7^th^ follow-up (after 15 months of enrolment)  □ 8^th^ follow-up (after 18 months of enrolment)  □ 9^th^ follow-up (after 21 months of enrolment)  □ 10^th^ follow-up (final follow-up) |
| A7 | Clinic code |  |

| **Section B: Home Blood Pressure Monitoring** | | | | |
| --- | --- | --- | --- | --- |
| **No.** | **Item** | **Yes** | **No** | **Remark** |
| B1 | Do you own a Blood Pressure monitor?  ***(If no, go to item C1 and advise to use home BP monitor)*** | □ | □ |  |
| B2 | Do you measure your BP at home regularly? | □ | □ |  |

| **Section C: Lifestyle modification for control of hypertension and global cardiovascular risk** | | | | | |
| --- | --- | --- | --- | --- | --- |
| **No.** | **Item** | **Aim** | **Yes** | **No** | **Remark** |
| C1 | Have you been having low salt diet? | Low salt diet | □ | □ |  |
| C2 | Have you been having a diet high in fruits and vegetables? | High content of fruits and vegetables in diet | □ | □ |  |
| C3 | Did you exercise regularly since our last conversation? | Exercise for 30 minutes for 5 or more days per week. | □ | □ |  |
| C4 | Did you drink excessively since our last conversation? | No more than 2 standard drinks per day.   - 2/3 small can of beer (220 ml) - 1 glass of wine (100ml) - 1 nip of spirit (30ml) | □ | □ |  |
| C5 | Did you smoke since our last conversation? | Stop smoking | □ | □ |  |
| C6 | ***If BMI ≥ 23.5 at baseline***, have you been trying to lose weight since our last conversation? | BMI < 23.5 | □ | □ |  |

| **Section D** | | | | | | |
| --- | --- | --- | --- | --- | --- | --- |
| **Hypertension medication adherence** | | | | | | |
| **No.** | **Item** | | | **Yes** | **No** | **Remark** |
| D1 | Was there any missed dose for your Hypertension medication since our last conversation?  **(If yes, go to next item D2. If No, go to D5.)** | | | □ | □ |  |
| D2 | ***If Yes***, how many times did you miss Hypertension medication? | | | | | Number of times: ______ |
| D3 | If poor adherence, what are the reasons? **(Tick all that apply)**  ***(If medication side effects, notify study coordinator for completing adverse events reporting form)*** | Medication Side effects | | | □ |  |
|  |  | Forgetfulness | | | □ |  |
|  |  | Misunderstanding about following regimen | | | □ |  |
|  |  | Changing schedule | | | □ |  |
| D4 | Other reasons, if any: | | | | | |
| **Lipid medication adherence** | | | | | | |
| **No.** | **Item** | | **Yes** | | **No** | **Remark** |
| D5 | Was there any missed dose for your Lipid medication since our last conversation?  **(If yes, go to next item D6. If No, go to section E.)** | | □ | | □ |  |
| D6 | ***If Yes***, how many times did you miss Lipid medication? | | | | | Number of times: ______ |
| D7 | If poor adherence, what is the reason? **(Tick all that apply)**  ***(If medication side effects, notify study coordinator for completing adverse events reporting form)*** | Medication Side effects | | | □ |  |
|  |  | Forgetfulness | | | □ |  |
|  |  | Misunderstanding about following regimen | | | □ |  |
|  |  | Changing schedule | | | □ |  |
| D8 | Other reasons, if any: | | | | | |

| **Section E** | | | | |
| --- | --- | --- | --- | --- |
| **Action** | | | | |
| **No.** | **Item** | **Reinforced the advice on:** | | **Remark** |
| E1 | Action taken during the telephone follow-up: | Advice on lifestyle modification strengthened | □ |  |
|  |  | Advice on medication adherence strengthened | □ |  |
|  |  | Medication side effect recorded | □ |  |
|  |  | Advice on home BP monitoring strengthened | □ |  |
| E2 | Other actions taken, if any: |  |  | |
| **Communication with Physician** | | | | |
| E3 | Additional action**,** if any, following the discussion with physician |  | | |
| E4 | Name and signature of the physician | **Name: ________________** **Signature: _______________**  **Date (dd/mm/yyyy): _ _ / _ _ / _ _ _ _** | | |
| E5 | Further communication with the participant following the discussion with physician |  | | |
| E6 | Dated signature of the nurse clinician or nurse after completion of actions | **Signature: _______________**  **Date (dd/mm/yyyy): _ _ / _ _ / _ _ _ _** | | |

# Section S3: Physician management checklist

| 1 | Study ID number |  |
| --- | --- | --- |
| 2 | Date (dd/mm/yyyy) | _ _ / _ _ / _ _ _ _ |
| 3 | Age of the participant |  |
| 4 | Signature of Physician |  |
| 5 | Clinic code |  |

| **Checklist to Identify High CVD Risk Subjects** | | | | |
| --- | --- | --- | --- | --- |
| **No.** | **Item** | **Yes** | **No** | **Remark** |
| 1 | Does participant have a CVD score identifying CVD risk **≥20%** over 10 years? | □ | □ |  |
| 2 | Does participant have **Diabetes**? | □ | □ |  |
| 3 | Does participant have **Left Ventricular Hypertrophy** (as evidenced by echocardiography or electrocardiography if participant has one)? | □ | □ |  |
| 4 | Does participant have **retinopathy**? | □ | □ |  |
| 5 | Does participant have **proteinuria** [ACR >34 mg/mmol of Cr; Urine albumin excretion >300 mg/day]? | □ | □ |  |
| 6 | Does participant have **renal disease** (Estimated GFR <60 ml/min/1.73m^2^)? | □ | □ |  |
| 7 | Does participant have **previous history of heart disease**? | □ | □ |  |
| 8 | Does participant have **previous history of stroke**? | □ | □ |  |

**CVD**, Cardiovascular Disease; **ACR**, Albumin-to-creatinine ratio; **Cr**, Creatinine

1. If any one of above items is “Yes”, then the participant belongs to high CVD risk group. Otherwise, s/he belongs to low/medium CVD risk group.

Based on the information above, the study participant has: **(Tick one below)**

□ **High CVD risk – skip to question B**  □ **Low/medium CVD risk – skip to question D**

1. If the participant belongs to High CVD risk group and falls in the age group 40-79, then was s/he prescribed fixed dose combination (FDC) drug and statin as per the study treatment algorithm? **(Tick one below)**

□ **Yes -> END**  □ **No – skip to question C**

1. If no, then what was the reason for not prescribing the FDC?

_________________________________________________________________________________

­­­­­­­ _________________________________________________________________________________

_________________________________________________________________________________

1. If the participant belongs to Low/medium CVD risk group having uncontrolled BP (SBP ≥160mm Hg or DBP ≥100 mm Hg) and falls in the age group 40-79, then was s/he prescribed fixed dose combination (FDC) drug and statin (only if serum LDL >4.1 mmol/l) as per the study treatment algorithm? **(Tick one below)**

□ **Yes -> END**  □ **No – skip to question E**

1. If no, then what was the reason for not prescribing the FDC?

_________________________________________________________________________________

­­­­­­­ _________________________________________________________________________________

_________________________________________________________________________________

# Section S4: Summary of intervention training

| **Health care provider** | **Training components** | **Duration** | **Trainer** |
| --- | --- | --- | --- |
| Physicians | Hypertension management for physicians consists of training on:   1. Definition and classification of hypertension 2. Guidelines for measurement of blood pressure 3. Evaluation of hypertensive individuals  - Clinical history - Physical exam - Laboratory testing - Assessing target organ damage  1. Cardiovascular risk assessment 2. Management of hypertension- treatment algorithm as per protocol  - Treating high-risk individuals using single-pill combination (SPC) antihypertensive and statins - Treating BP to target in all hypertensives as per algorithm  1. Case-based scenarios to practice hypertension management algorithm | Initial training 2-hours (two 1-hour lunch time CME sessions)  Refresher 1-hour annually | Hypertension Specialist- Nephrologist |
| Nurses | Motivational interviewing (MI) training curriculum for nurses includes:   1. Definition and Theoretical basis 2. Evidence & spirit 3. Principles of MI  - Express empathy - Develop discrepancy - Roll with resistance - Support self-efficacy  1. Skills for MI – OARS  - Asking open-ended questions (O) - Affirming (A) - Reflective listening (R) - Summarizing (S)  1. Stages of change 2. Change talk – DARN-C  - Desire (D) - Ability (A) - Reasons (R) - Need (N) - Commitment language (C)  1. The decisional-balance sheet (exploring ambivalence) 2. Importance and confidence rulers 3. Who might benefit from MI & contraindications to MI 4. Case studies and demos | 1-day training (initial)  Half-day training (refresher) annually | Clinical psychologist |

# **Section S5: Antihypertensive treatment algorithm ^1,2,3,4^**


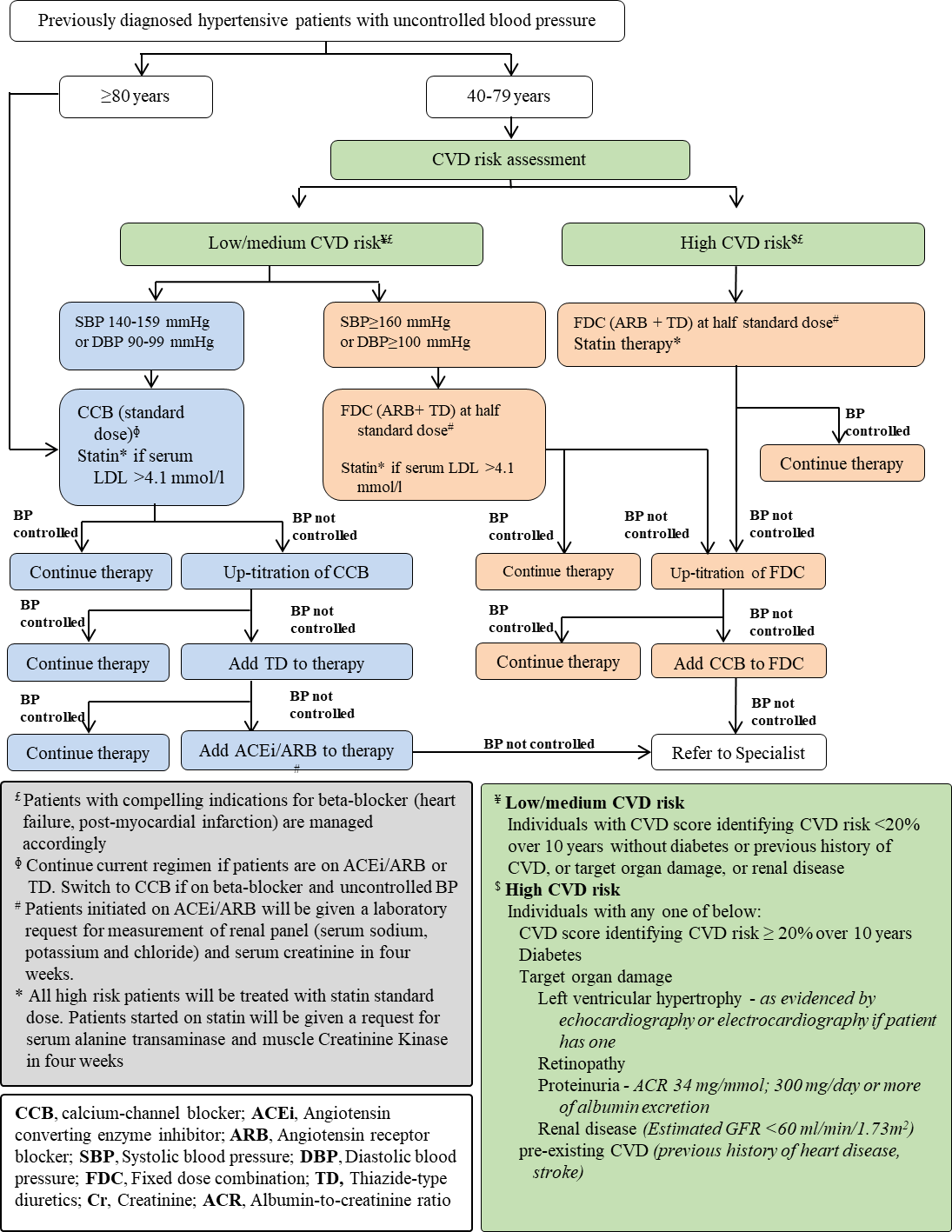


The treatment algorithm required all participants to undergo cardiovascular risk stratification which is embedded in the polyclinic electronic medical system. Individuals with a Framingham CVD score indicating a risk of acute coronary heart disease (CHD) of 20% or more over ten years, diabetes, target organ damage, or pre-existing CVD were categorized as high risk. All other hypertensive individuals were categorized as low/medium risk. For high-risk participants, the SPC with angiotensin II receptor blocker (ARB) and a diuretic was recommended at half-standard each and titrated to a full dose if BP remains uncontrolled. Statins were also initiated for LDL-C < 2.6 mmol/L (< 100 mg/dl), as per the algorithm. For participants at low/medium risk, monotherapy with calcium channel blockers, ACEIs (preferably in those less than 55 years) or thiazide diuretics (preferably in those aged 55 years and older) was initiated at half-standard dose and up-titrated as necessary. Additional agents belonging to either of the drug classes were added if BP were uncontrolled. The target BP was <140/90 mmHg. However, the target BP for hypertensive individuals with proteinuria or pre-existing CVD was < 130/80 mmHg. The physicians completed a standardized management checklist.

# Section S6: Statistical methods for sensitivity for systolic blood pressure (BP)

The following sensitivity analyses were performed for the primary outcome of systolic BP.

**Sensitivity Analysis 1: Per protocol analysis.**

A Per Protocol analysis was conducted as sensitivity analysis for the outcome of systolic BP. Participants who received all components of the multicomponent intervention were included in the intervention group. Since intervention fidelity to receiving are by trained physician, motivational conversation, and telephone follow-ups were more than 95% no participants were excluded for not receiving these components as per protocol. However, those who were high CVD risk at baseline and did not receive single pill combination medication as per the multi-component intervention (MCI) algorithm (n=73), were excluded from the intervention group in the Per Protocol analysis.

**Sensitivity Analysis 2: Primary analysis on patients completing the 24-month follow-up.**

This analysis was conducted on the primary outcome of systolic BP at 24 months for participants completing the final 2-year follow up (n=766) only. Participants who did not complete the final follow up (n=150) were excluded from the analysis.

**Sensitivity Analysis 3: Systolic BP outcome after adjusting for clinically important variables.**

This analysis for this ITT was conducted for the outcome of systolic BP at 24 months at the participant level after adjusting for important and clinically meaningful baseline characteristics as covariates. The characteristics included as covariates in adjustments were age, gender, baseline waist circumference, diabetes and 10-year FRS CVD Risk score at baseline.

**Sensitivity Analysis 4: Systolic BP outcome after imputing missing values at 12- & 24-month.**

ITT after multiple imputation, in which missing values of 1 (15.0%) and 2 year (16.4%) systolic BP were replaced through a process of multiple imputation. Variables were included in the imputation model if they were risk factors for hypertension, or were correlated with systolic BP years 1 or 2 or its missingness. Therefore, the imputation model included baseline age, gender, intervention group, waist circumference, 10-year FRS CVD Risk score, and systolic BP at baseline. The resultant dataset allowed us to use data from all 916 individuals randomized. Twenty multiply-imputed datasets were created using fully conditional specification (FCS) predictive mean matching methods under the missing at random assumptions.^5^ After imputation, each of the 20 datasets was analyzed using a MMRM model similar to the approach used for the non-imputed dataset. The parameter estimates obtained from each dataset were pooled for inference.

**Sensitivity Analysis 5: Systolic BP outcome restricted to participants followed before COVD-19.**

This analysis was conducted to assess the intervention effect on the primary outcome before the world health organization declared COVID-19 as pandemic on 12 March 2020. The systolic BP readings of the participants who completed the 2-year follow-up on or after 12 March (n=72, intervention – 62, and usual care - 10) were excluded from the analysis.

All the analyses were performed in SAS software (version 9.4) using the GLIMMIX procedure.

# Section S7: Program delivery cost estimation – methods, assumptions, and data sources

We organised the within trial intervention costs into 6 main categories. **Table 1** summarizes the key cost intervention components by activity and **Table 2** summarizes the key cost components of the intervention by year. The activities and cost assignment related to each category is as follows.

**A. Incremental Cost of Intervention**

**1. Admin and oversight**

Labor cost: Includes time spent by nurses in planning, coordinating, and monitoring motivational conversation delivery, and time spent by site PI/physicians in meetings/discussions.

Based on senior nurses’ and general practitioners’ salaries^6-7^ and the man-hours spent, 14.4% of total manpower costs were assigned for admin and oversight activities spread over the 2 years. Admin and oversight duties for nurses include listing and rostering subjects for telephone calls (monthly), filling up checklists, documentation of the motivational conversations in electronic medical records (EMR), coordinating transfer of checklists and tracking monthly task completion by nurses. Admin and oversight time of 1 hour every two months (12 hours for 2 years) allocated for site PI/physician and site Co-I/senior nurse time for meetings/discussions to assess progress of intervention delivery, and discussion with nurses and physicians.

**2. Training nurses in motivational conversation and telephone follow-ups**

Trainer fees: External trainers, specialist psychologists from an accredited counselling centre, conducted all training sessions at a central venue. Cost of contracted external trainers with initial full-day (8-hours) training was SGD 3600 per session of 10-12 attendees, and refresher half-day (4-hours) training at SGD 1800 per session of 6-7 attendees (year 2 half day training SGD 2000 per session). Year 1 cost includes 3 initial and 3 refresher training sessions (at end of year 1), and year 2 cost includes 2 refresher training sessions.

In kind cost: Includes cost of refreshments provided during training sessions valued at market rates. Year 1 cost for 6 sessions and year 2 cost for 2 sessions.

Opportunity cost for nurse trainees: The time spent attending the training sessions is part of the registered nurses’ FTE^8^ which is included as opportunities costs and valued at market rates.

**3. Training physicians**

Trainer fees: Includes per diem paid to trainer at the rate of SGD 300 per hour. Each training session limited to 1 hour during lunch time in the respective clinics. Year 1 cost includes 2 initial training (1 hour each session of 10-15 attendees) at baseline and one refresher (1 hour) session of 8-10 attendees in each clinic totalling 12 hours. Year 2 cost includes one refresher (1 hour) session of 8-10 attendees in each clinic totalling 4 hours.

Materials and supplies: Cost of printing training manuals, checklists and algorithm displays incurred once at baseline.

In kind cost: Includes cost of lunch and transportation for trainers valued at market rates.

Cost of lunch provided to all participants during training: Year 1 includes 12 sessions, and year 2 includes 4 sessions.

Transportation costs for trainers to and from form the venue: Year 1 includes 12 trips, and year 2 includes 4 trips.

Opportunity cost for physician trainees: The time spent attending the training sessions is part of the physicians’ FTE^7^ which is included as opportunities costs and valued at market rates.

**4. Implementation of MC and telephone follow-ups**

Labor cost: Includes time spent by nurses delivering face-to-face MC in-clinic (1 session at baseline) and telephone follow-up calls (10 calls per subject). For labor, the remaining 85.6% of total manpower cost was assigned towards implementation of MC and telephone follow-up for the 2 years.

Year 1 costs include time spent by nurses for one face-to-face MC session provided to individuals classified as high-risk for cardiovascular disease (CVD) provided at baseline and telephone follow-up calls performed during year 1 (total 6 calls). Approximately 50% of high-risk intervention subjects (200 subjects) received face-to-face MC in-clinic lasting on an average between 20-30 minutes per subject. In addition, nurses provided telephone follow-up to all intervention subjects (400 subjects) and made six calls per subject in year-1 with each call averaging 5.35 minutes.

Year 2 costs include nurse’s time for providing 4 telephone follow-up calls per subject for all intervention subjects (400 subjects) with each call averaging 5.35 minutes.

Materials and supplies: Costs incurred for printing of checklists was spread over 2 years. Air time cost of telephone calls for the two-year period: Year 1 airtime for 6 calls per participant and year-2 airtime for 4 calls per participant.

**5. General practitioner visit costs**

Visit cost: The number of general practitioner visits is based on the number of times patient visited the clinic during the study. Taking the incremental difference in the number visits between intervention and usual care over the two years of the study which is 0.345, we multiplied by all intervention subjects (400 subjects) and cost for each visit (SGD 51.50)^9^ to get the total visit cost. For years 1 and 2 each, we divide the incremental difference in the number of visits between intervention and usual care over the two years of the study equally, which is 0.173 each.

**6. Single-Pill Combination (SPC) subsidy**

Materials and supplies: 50% subsidy for SPC antihypertensive medication provided for 2 year’s cost of 0.20 per unit for Hyzaar standard dose and 0.30 per unit for Hyzaar forte. Total cost was spread over 2 years of intervention.

**7. Laboratory tests related to SPC**

Materials and supplies: Cost of safety lab test for individuals prescribed SPC (Hyzaar) and/or statins done once after initiation of SPC at SGD 51 per test constitutes Year-1 cost. The labs include serum sodium, potassium, chloride, and creatine kinase.

Aggregating all these costs, the cost of intervention per participant per year would be SGD 227.65.

**8. Excess costs for other anti-hypertensives and lipid lowering medications**

Materials and supplies: The total cost of antihypertensive medications and lipid lowering medications was obtained from clinic pharmacy data which includes the subsidy and the co-payment paid by the patients. The difference in the total costs among all subjects between the intervention and usual care group was computed over the 2-year follow-up assessment. The excess cost in the intervention group relative to usual care was used in the cost analysis. For years 1 and 2, we divide the total excess cost over the two years equally.

**Box 1: Key cost components of SingHypertension intervention by activity**

| **Activity** | **Key cost components to be tracked** |
| --- | --- |
| Admin and oversight | Labor:   - Time spent by nurses in planning, coordinating, and monitoring motivational conversation delivery (11.2% of total labor costs) - Time spent by site PI/physicians in meetings/discussions (3.2% of total labor costs) |
| Training nurses in motivational conversation (MC) & telephone follow up | Trainer fees:   - Cost of contracted external trainers - Includes initial and refresher trainings   In kind cost:   - Refreshments provided during trainings   Opportunity cost for nurse trainees:   - Time spent by nurses attending training session |
| Training physicians | Trainer fees:   - Per diem paid to trainers - Includes initial and refresher trainings   Materials and supplies:   - Training material: printing of manuals, checklists and algorithms   In kind cost:   - Refreshments provided during trainings - Transportation provided for trainers   Opportunity cost for physician trainees:   - Time spent by nurses attending training session |
| Implementation of MC & telephone follow up | Labor (85.6% of total labor costs):   - Time spent by nurses delivering MC in-clinic (1 session) - Time spent by nurses in telephone follow-up (10 sessions)   Materials and supplies:   - Printing of checklists - Air time cost of telephone calls |
| General practitioner visit costs | Visit costs:   - Incremental number of non-resident general practitioner visits between intervention and usual care groups multiplied by the cost of each visit. The number of non-resident general practitioner visits is based on the number of times medication was dispensed to the patient at the clinic during the study. |
| Lab tests related to SPC | Materials and supplies   - Costs of safety lab tests |
| Excess costs for other anti-hypertensives, statins | Materials and supplies   - Excess costs of other anti-hypertensive and statin medications borne by intervention group |

**Box 2: Key cost components of SingHypertension intervention by year**

| **Activity** | **Cost allocation** | **Year-1 cost** | **Year-2 cost** |
| --- | --- | --- | --- |
| Admin and oversight | Labor (14.4% of total labor costs) | - Oversight provided by one nurse manager at 0.03 FTE in each clinic for planning, coordinating, and monitoring motivational conversation delivery for nurses - listing and rostering subjects for telephone calls (monthly) - filling up checklists - documentation of the motivational conversations in electronic medical records (EMR) - coordinating transfer of checklists - tracking monthly task completion by nurses - assess progress of intervention delivery and discussions with nurses (1 hour every two months) - Oversight provided by one site PI/physician at 0.003 FTE in each clinic to assess progress of intervention delivery and discussions with nurses (1 hour every two months) | Same as year-1 |
| Training nurses in motivational conversation (MC) & telephone follow up | Trainer fees | - Contracted training from specialist psychologists from an accredited counselling center conducted at a central venue. To includes nurses from all clinics, with each nurse attending one of three initial training (10-12 attendees each session) and attending one of three refresher training sessions (6-7 attendees each session) - 3 initial (each with full-day [8 hours] training session) and 3 refresher (each with half-day [4 hours] training session) | - Contracted training from specialist psychologists from an accredited counselling center conducted at central venue. To include nurses from all clinics, with each nurse attending one of 2 refresher training (6-7 attendees each session) - 2 refresher (each with half-day [4 hours] training session) |
|  | In kind cost | - Refreshments provided during trainings- 6 sessions (3 initial training and 3 refresher training sessions) | - Refreshments provided during trainings -2 sessions (2 refresher training sessions) |
|  | Opportunity cost for nurse trainees | - Time spent during trainings - 6 sessions (3 initial training for 10-12 trainees each and 3 refresher training sessions for 6-7 trainees each) | - Time spent during trainings - 2 sessions (2 refresher training sessions for 6-7 trainees each) |
| Training physicians | Trainer fees | - Trainer fees incurred per hour of training in each clinic - Physician training conducted in each clinic - 2 hours allocated for each initial training and 1 hour for each refresher training - 1 initial (each with two 1-hour training sessions for 10-15 attendees) and 1 refresher (each with one 1hour training session for 8-10 attendees) in each clinic. Four clinics in total. | - Trainer fees incurred per hour of training in each clinic - Physician training conducted in each clinic - 1 hour for each refresher training - 1 refresher (each with one 1hour training session for 8-10 attendees) in each clinic. Four clinics in total. |
|  | Materials and supplies | - Training material: printing of manuals, checklists and algorithms | NIL |
|  | In kind cost | - Refreshments provided during trainings-12 sessions - Transportation provided for trainers-12 trips | - Refreshments provided during training -4 sessions - Transportation provided for trainers -4 trips |
|  | Opportunity cost for physician trainees | - Time spent during trainings-12 sessions [1 initial (each with two 1-hour training sessions for 10-15 trainees) and 1 refresher (each with one 1hour training session for 8-10 trainees) in each clinic. Four clinics in total.] | - Time spent during trainings- 4 sessions [1 refresher (each with one 1hour training session for 8-10 trainees) in each clinic. Four clinics in total.] |
| Implementation of MC & telephone follow up | Labor (85.6% of total labor costs) | - Time spent by nurses delivering MC in-clinic (1 session) at baseline approximately 20-30 min per participant - Time spent by nurses in telephone follow-up, average of 5.35 minutes per call (6 calls per participant) | - Time spent by nurses in telephone follow-up, average of 5.35 minutes per call (4 calls per participant) |
|  | Materials and supplies | - Printing of checklists - Air time cost of telephone calls (6 calls per participant) | - Printing of checklists - Air time cost of telephone calls (4 calls per participant) |
| General practitioner visit costs | Visit costs | - Incremental difference in the number of general practitioner visits between intervention and usual care over a year of the study at 0.173 | Same as year-1 |
| Single pill combination (SPC) subsidy | Materials and supplies | - Cost of subsidy for SPC medication per participant per day for Hyzaar standard dose and for Hyzaar forte dose | Same as year-1 |
| Lab tests related to SPC | Materials and supplies | - Cost of safety lab test for participants initiated on SPC at baseline | NIL |
| Excess costs for other anti-hypertensives, statins | Materials and supplies | - Excess costs of other anti-hypertensive and statin medications borne by intervention group | Same as year-1 |

For Box 1 and Box 2, all costs were collected in local currency, converted to U.S. dollars using exchange rates as of 1 Mar 2021.

# Section S8: Supplementary tables

## Table A. Baseline Characteristics

|  |  | **All clinics (N=916)** |  |  |
| --- | --- | --- | --- | --- |
| **Characteristics ^a^** | **Total (N=916, 8 clinics)** | **Multicomponent Intervention (N=447, 4 clinics)** | **Usual Care (N=469, 4 clinics)** | **P value** |
| Age (years), Mean (SD) | 64.5 (9.8) | 63.0 (9.7) | 65.9 (9.7) | 0.41 |
| Female, n (%) | 454 (49.6) | 203 (45.4) | 251 (53.5) | 0.13 |
| Ethnicity, n (%) |  |  |  | 0.88 |
| Chinese | 673 (73.5) | 334 (74.7) | 339 (72.3) |  |
| Malay | 122 (13.3) | 57 (12.8) | 65 (13.9) |  |
| Indian | 89 (9.7) | 36 (8.1) | 53 (11.3) |  |
| Other | 32 (3.5) | 20 (4.5) | 12 (2.6) |  |
| Housing type, n (%) ^b^ |  |  |  | 0.27 |
| very low income gov’t flat (1-3 rooms) | 252 (27.5) | 97 (21.7) | 155 (33.0) |  |
| Govt. housing 4 rooms or higher-tiered or private condominium | 659 (71.9) | 350 (78.3) | 309 (65.9) |  |
| Own subsidized flat/condominium, n (%) | 778 (84.9) | 380 (85.0) | 398 (84.9) | 0.83 |
| Education Level, n (%) ^c^ |  |  |  | 0.22 |
| Primary or lower | 245 (26.7) | 95 (21.3) | 150 (32.0) |  |
| Secondary or higher | 670 (73.1) | 351 (78.5) | 319 (68.0) |  |
| Currently employed, n (%) | 445 (48.6) | 240 (53.7) | 205 (43.7) | 0.33 |
| Monthly income, n (%) |  |  |  | 0.66 |
| 1. < 2000 SGD | 272 (29.7) | 131 (29.3) | 141 (30.1) |  |
| 2. 2000 to < 4000 SGD | 93 (10.2) | 46 (10.3) | 47 (10.0) |  |
| 3. > 4000 SGD | 169 (18.4) | 107 (23.9) | 62 (13.2) |  |
| 4. Prefer not to say | 381 (41.6) | 162 (36.2) | 219 (46.7) |  |
| Currently married, n (%) | 678 (74.0) | 349 (78.1) | 329 (70.1) | 0.08 |
| Overweight or obese, n (%) ^d^ | 671 (73.3) | 324 (72.5) | 347 (74.0) | 0.80 |
| High waist circumference, n (%) ^e^ | 658 (71.8) | 308 (68.9) | 350 (74.6) | 0.39 |
| Chronic diseases |  |  |  |  |
| Self-reported heart disease, n (%) | 92 (10.0) | 33 (7.4) | 59 (12.6) | 0.24 |
| Self-reported stroke, n (%) | 37 (4.0) | 20 (4.5) | 17 (3.6) | 0.62 |
| Diabetes, n (%) ^f^ | 314 (34.3) | 138 (30.9) | 176 (37.5) | 0.45 |
| Chronic kidney disease, n (%) ^g^ | 351 (38.3) | 165 (36.9) | 186 (39.7) | 0.56 |
| Any of above chronic diseases, n (%) ^h^ | 548 (59.8) | 245 (54.8) | 303 (64.6) | 0.10 |
| Multi-morbidity (> 2 of above chronic diseases), n (%) ^i^ | 197 (21.5) | 86 (19.2) | 111 (23.7) | 0.38 |
| High CVD risk ^j^ | 479 (52.3) | 215 (48.1) | 264 (56.3) | 0.14 |
| Systolic BP, Mean (SD), mm Hg | 149.3 (13.0) | 148.4 (11.8) | 150.1 (14.0) | 0.30 |
| Diastolic BP, Mean (SD), mm Hg | 88.3 (9.8) | 89.4 (9.2) | 87.3 (10.2) | 0.26 |
| Poorly controlled BP, n (%) ^k^ | 48 (5.2) | 22 (4.9) | 26 (5.5) | 0.73 |
| Self-reported health status on EQ-5D-5L VAS, Mean (SD) ^l^ | 75.4 (13.4) | 77.0 (12.3) | 74.0 (14.2) | 0.48 |
| EQ-5D-5L utility index, Mean (SD) ^m^ | 0.93 (0.08) | 0.94 (0.08) | 0.92 (0.09) | 0.50 |
| Smoking Status, n (%) |  |  |  | 0.21 |
| Currently Smoking | 78 (8.5) | 40 (8.9) | 38 (8.1) |  |
| Former Smoking | 116 (12.7) | 70 (15.7) | 46 (9.8) |  |
| Non-smokers | 722 (78.8) | 337 (75.4) | 385 (82.1) |  |
| Physical activity, Mean (SD), log MET-min/week ^n^ | 6.1 (2.4) | 6.3 (2.4) | 6.0 (2.3) | 0.28 |
| Frequency of fruits and vegetables consumption per week, n (%) |  |  |  | 0.39 |
| Fruits/uncooked Veg < 4 times per day | 912 (99.6) | 444 (99.3) | 468 (99.8) |  |
| Fruits/uncooked Veg 4 times or higher per day | 4 (0.4) | 3 (0.7) | 1 (0.2) |  |
| Sodium intake, mean (SD), g/day ^o^ | 4.3 (2.2) | 4.2 (1.5) | 4.3 (2.7) | 0.79 |
| Log of urine spot albumin-to-creatinine ratio (mg/mmol), Mean (SD) | 1.39 (0.96) | 1.34 (0.91) | 1.43 (1.0) | 0.31 |
| Fasting blood glucose (mmol/L), Mean (SD), mmol/L | 6.0 (1.6) | 5.9 (1.5) | 6.1 (1.8) | 0.55 |
| Total cholesterol, Mean (SD), mmol/L | 4.6 (0.9) | 4.7 (0.9) | 4.6 (0.9) | 0.30 |
| HDL cholesterol, Mean (SD), mmol/L | 1.4 (0.4) | 1.4 (0.4) | 1.4 (0.4) | 0.67 |
| LDL cholesterol, Mean (SD), mmol/L | 2.5 (0.8) | 2.6 (0.8) | 2.5 (0.7) | 0.27 |
| Triglycerides, Mean (SD), mmol/L | 1.5 (0.8) | 1.5 (0.8) | 1.4 (0.7) | 0.38 |
| Glycated Hemoglobin, Mean (SD), % ^p^ | 7.5 (1.3) | 7.5 (1.3) | 7.5 (1.2) | 0.69 |
| 10-year calculated CVD Framingham risk score % (FRS), mean (SD) | 22.2 (7.6) | 21.7 (7.6) | 22.7 (7.5) | 0.34 |
| CVD risk score categories by FRS, n (%) ^q^ |  |  |  | 0.17 |
| Low (< 10%) | 51 (5.6) | 27 (6.0) | 24 (5.1) |  |
| Medium (10 to < 20 %) | 311 (34.0) | 171 (38.3) | 140 (29.9) |  |
| High (> 20%) | 532 (58.1) | 245 (54.8) | 287 (61.2) |  |
| 10-year integrated CVD (ICVD) risk score %, Mean (SD) ^r^ | 14.7 (12.0) | 12.7 (10.5) | 16.6 (12.9) | 0.09 |
| Taken prescribed anti-hypertensive medication today or in last 2 days (self-reported) | 868 (98.3) | 432 (98.6) | 436 (98.0) | 0.29 |
| Currently on antihypertensive medication, n (%) ^s^ |  |  |  | 0.29 |
| 0 | 26 (2.8) | 9 (2.0) | 17 (3.6) |  |
| 1 | 408 (44.5) | 208 (46.5) | 200 (42.6) |  |
| 2 | 315 (34.4) | 158 (35.3) | 157 (33.5) |  |
| 3 or more | 154 (16.8) | 70 (15.7) | 84 (17.9) |  |
| Mean (SD) | 1.7 (0.9) | 1.7 (0.8) | 1.7 (0.9) | 0.48 |
| Single-pill combination antihypertensive medications, n (%) | 24 (2.6) | 14 (3.1) | 10 (2.1) | 0.41 |
| Class of antihypertensive medications, n (%) |  |  |  |  |
| CCB | 566 (61.8) | 275 (61.5) | 291 (62) | 0.90 |
| RAAS inhibitors | 484 (52.8) | 245 (54.8) | 239 (51) | 0.36 |
| BB | 346 (37.8) | 166 (37.1) | 180 (38.4) | 0.80 |
| Diuretics | 123 (13.4) | 55 (12.3) | 68 (14.5) | 0.45 |
| Others | 9 (1) | 1 (0.2) | 8 (1.7) | 0.27 |
| Cholesterol lowering medications, n (%) | 641 (71.0) | 307 (69.0) | 334 (72.9) | 0.62 |
| Number of oral antidiabetic medications, mean (SD)^t^ | 1.5 (1.1) | 1.6 (1.1) | 1.4 (1.1) | 0.58 |

Abbreviations: BB, beta blocker; BP, blood pressure; CCB, calcium channel blocker; CVD, cardiovascular diseases; DBP, diastolic blood pressure; EQ-5D-5L, EuroQol 5-Dimension 5-Level questionnaire; MCI, multicomponent intervention; OR, odds ratio; RAAS, Renin-angiotensin-aldosterone system; SBP, systolic blood pressure; SD, standard deviation; SGD, Singapore dollar; SPC, single pill combination; VAS, visual analogue scale.

^a^ Generalized mixed effects model was used to compare baseline variables accounting for cluster random effects for clinics.

^b^ For Housing type, 5 participants had missing responses in usual care.

^c^ For Education level, 1 participant in intervention group responded as ‘Don’t know/can’t remember’ which was treated as missing.

^d^ Defined as body mass index (BMI) > 23.5 kg/m^2^ per Asian cut-offs.

^e^ High waist circumference was defined as waist circumference of > 90 cm in males and > 80 cm in females.

^f^ Diabetes is defined as physician diagnosed or fasting blood glucose (FBG) > 7 mmol/L or glycated hemoglobin (HbA1c) > 6.5 %.

^g^ Chronic kidney disease is defined as estimated glomerular filtration rate (eGFR) < 60 ml/min/1.73m^2^ or urine albumin-to-creatinine ratio (ACR) > 3 mg/mmol.

^h^ At least one co-morbid chronic diseases of diabetes, chronic kidney disease, self-reported heart disease, and self-reported stroke.

^i^ More than two co-morbid chronic diseases of diabetes, chronic kidney disease, self-reported heart disease, and self-reported stroke.

^j^ Defined using the standardized physician management checklist developed for the study. The participant is categorized as high CVD risk if any of the following criteria is met: high CVD risk by Framingham Risk Score (FRS) adapted for Singapore >20, diabetes, albumin to creatinine ratio (ACR) >34mg/mmol, eGFR <60 ml/min/1.73m², physician diagnosed heart disease, and self-reported stroke.

^k^ Defined as systolic blood pressure > 160 mmHg and diastolic blood pressure > 100 mmHg.

^l^ Health status was reported on a scale of 0 (worst imaginable health) to 100 (best imaginable health) using EQ-5D-5L VAS, where higher values indicate better health.

^m^ EQ-5D-5L utility index was calculated using the Indonesian value set. It ranges from -0.865 (worst state) to 1 (full health), where higher values indicate higher health utility.

^n^ Physical activity was assessed using the International Physical Activity Questionnaire (IPAQ) score.

The IPAQ Group, 2005. Guidelines for data processing and analysis of the international physical activity questionnaire (IPAQ) - short and long forms.

For Physical activity level, 7 participants had missing responses in MCI and 1 in Usual Care.

^o^ The sodium intake is calculated using urine spot sodium and creatinine using Kawasaki formula.

Kawasaki T, Itoh K, Uezono K, Sasaki H. A simple method for estimating 24 h urinary sodium and potassium excretion from second morning voiding urine specimen in adults. *Clin Exp Pharmacol Physiol*. 1993;20(1):7-14.

^p^ CVD risk categories based on Glycated hemoglobin (Hb) is reported for participants who has diabetes at baseline by American Diabetes Association (ADA) criteria. 314 participants were diabetic, 138 in MCI and 176 in Usual Care.

For glycated Hb, 15 participants had missing values, 4 in MCI and 11 in Usual Care.

^q^ CVD risk categories based on the 10-year Framingham risk score.

For CVD risk score, 22 participants had missing CVD scores, 4 in MCI and 18 in Usual Care.

^r^ A 10-year integrated cardiovascular disease (ICVD) risk score for coronary events and ischemic stroke was computed using the equation developed for the Chinese adults. Wu Y et al, 2006 Estimation of 10-Year Risk of Fatal and Nonfatal Ischemic Cardiovascular Diseases in Chinese Adults. *Circulation*. 2006; 114:2217-2225.

^s^ For Currently on antihypertensive medication, there were 13 participants with missing pharmacy data.

^t^ Among participants with diabetes at baseline by ADA criteria. 314 participants were diabetic, 138 in MCI and 176 in Usual Care.

## Table B. Implementation and Intervention Fidelity

|  | **Overall (4 clinics)**  **(N=447)** | | **Clinic 1**  **(N=111)** | | **Clinic 2**  **(N=107)** | | **Clinic 3**  **(N=118)** | | **Clinic 4**  **(N=111)** | |  |
| --- | --- | --- | --- | --- | --- | --- | --- | --- | --- | --- | --- |
|  | **n/N** | **% (95% CI)** | **n/N** | **% (95% CI)** | **n/N** | **% (95% CI)** | **n/N** | **% (95% CI)** | **n/N** | **% (95% CI)** | |
| **Implementation fidelity** |  |  |  |  |  |  |  |  |  |  | |
| Eligible physicians trained in hypertension management algorithm ^a^ | 42/42 | 100.0 (91.6, 100.0) | 14/14 | 100.0 (76.8, 100.0) | 9/9 | 100.0 (66.4, 100.0) | 11/11 | 100.0 (71.5, 100.0) | 8/8 | 100.0 (63.1, 100.0) | |
| Eligible nurses trained in motivational conversation ^b^ | 23/23 | 100.0 (85.2, 100.0) | 7/7 | 100.0 (59.0, 100.0) | 5/5 | 100.0 (47.8, 100.0) | 7/7 | 100.0 (59.0, 100.0) | 4/4 | 100.0 (39.8, 100.0) | |
| **Intervention fidelity** |  |  |  |  |  |  |  |  |  |  | |
| Physician checklists completed ^c^ | 447/447 | 100.0 (99.2, 100.0) | 111/111 | 100.0 (96.7, 100.0) | 107/107 | 100.0 (96.6, 100.0) | 118/118 | 100.0 (96.9, 100.0) | 111/111 | 100.0 (96.7, 100.0) | |
| Participants ever received nurses’ telephone follow-up based on checklists ^d^ | 445/447 | 99.6 (98.4, 99.9) | 110/111 | 99.1 (95.1, 100.0) | 106/107 | 99.1 (94.9, 100.0) | 118/118 | 100.0 (96.9, 100.0) | 111/111 | 100.0 (96.7, 100.0) | |
| Average number of nurses’ telephone follow-up completed per participant based on checklists ^e^ | 447/447 | 7.2 (7.0, 7.4) | 111/111 | 7.0 (6.6, 7.4) | 107/107 | 7.1 (6.7, 7.6) | 118/118 | 7.8 (7.5, 8.2) | 111/111 | 6.8 (6.4, 7.2) | |
| Average duration of nurses’ telephone follow-up completed per follow-up | 447/447 | 5.4 (0.0, 12.4) | 111/111 | 4.0 (0.0, 9.4) | 107/107 | 5.5 (0.0, 11.6) | 118/118 | 3.7 (0.0, 8.6) | 111/111 | 8.6 (0.0, 15.8) | |
| High-risk participants received motivational conversation in-clinic ^f^ | 190/201 | 94.5 (90.4, 97.2) | 45/48 | 93.8 (82.8, 98.7) | 37/40 | 92.5 (79.6, 98.4) | 47/49 | 95.9 (86.0, 99.5) | 61/64 | 95.3 (86.9, 99.0) | |
| High-risk participants prescribed SPC ^g^ | 128/201 | 63.7 (56.6, 70.3) | 25/48 | 52.1 (37.2, 66.7) | 22/40 | 55.0 (38.5, 70.7) | 33/49 | 67.3 (52.5, 80.1) | 48/64 | 75.0 (62.6, 85.0) | |
| Single-pill combination antihypertensive medication subsidy ^h^ | 124/128 | 96.9 (92.3, 99.1) | 24/25 | 96.0 (79.6, 99.9) | 22/22 | 100.0 (84.6, 100.0) | 31/33 | 93.9 (79.8, 99.3) | 47/48 | 97.9 (88.9, 99.9) | |

Abbreviations: CVD, cardiovascular diseases; SPC, single-pill combination; MCI, multicomponent intervention, n=number participated, N=number eligible.

^a^ Eligible physicians refers to all physicians assigned to participate in the study from the 4 MCI clinics.

^b^ Eligible nurses refers to all nurses assigned to participate in the study from the 4 MCI clinics.

^c^ Number of physician checklists completed and denominator is total number of participants in the MCI group.

^d^ Proportion of participants who received at least one nurse’s telephone follow-up based on checklists received among the participants in MCI group.

^e^ Total number of completed nurse’s telephone follow-up checklists in the 2-year period per participant among those who completed at least one checklist over 2-years.

^f^ Number given motivational conversation in-clinic and denominator is number of individuals identified as high-risk for CVD and age < 80 years.

^g^ Total number of completed nurse’s telephone follow-up checklists in the 2-year period per participant among those who completed at least one checklist over 2-years.

^h^ Number received medication subsidy for SPC (Hyzaar) and denominator is number of individuals prescribed any single pill combination antihypertensive and eligible for SPC as per the checklist.

## **Table C. Intervention Effect on Secondary Outcomes per ITT** ^a^

|  | **n** | **Multicomponent**  **Intervention**  **(N=447, 4 clinics)** | **n** | **Usual care**  **(N=469, 4 clinics)** | **Adjusted Means Difference**  **/OR ^b^ (95% CI)** | **P-value** |
| --- | --- | --- | --- | --- | --- | --- |
| **Various thresholds of BP control** |  |  |  |  |  |  |
| BP controlled to target per study algorithm, % (95% CI) ^c^ |  |  |  |  |  |  |
| 12 months | 372 | 47.5 (40.1, 55.0) | 407 | 41.8 (36.8, 46.9) | 1.26 (0.87, 1.84) |  |
| 24 months | 384 | 54.6 (47.1, 61.9) | 382 | 43.8 (38.7, 49.0) | 1.55 (1.06, 2.25) | **0.02** |
| BP controlled to target per study algorithm or ≥ 5 mmHg reduction in SBP, % (95% CI) |  |  |  |  |  |  |
| 12 months | 372 | 74.0 (67.9, 79.3) | 407 | 65.9 (59.3, 72.0) | 1.47 (0.96, 2.26) |  |
| 24 months | 384 | 76.3 (70.4, 81.4) | 382 | 66.8 (60.1, 72.9) | 1.60 (1.04, 2.47) | **0.03** |
| Uncontrolled blood pressure (SBP > 140 mmHg or DBP > 90 mmHg), % (95% CI) ^c^ |  |  |  |  |  |  |
| 12 months | 372 | 45.5 (40.3, 50.9) | 407 | 51 (45.9, 56) | 0.8 (0.59, 1.09) |  |
| 24 months | 384 | 38.7 (33.7, 43.9) | 382 | 48.8 (43.6, 54) | 0.66 (0.49, 0.9) | **0.01** |
| BP poorly controlled (SBP ≥ 160 mmHg and DBP ≥ 100 mmHg), % (95% CI) |  |  |  |  |  |  |
| 12 months | 372 | 0.8 (0.3, 2.3) | 407 | 2.8 (1.1, 6.7) | 0.27 (0.06, 1.21) |  |
| 24 months | 384 | 1.9 (0.9, 4.0) | 382 | 2.4 (0.9, 6.1) | 0.80 (0.22, 2.91) | 0.74 |
| **Lifestyle measures** |  |  |  |  |  |  |
| Physical activity (change in log MET/min per week from baseline), mean (95% CI) ^d^ |  |  |  |  |  |  |
| 12 months | 372 | 1.0 (0.4, 1.6) | 407 | 0.7 (0.1, 1.3) | 0.27 (-0.41, 0.95) |  |
| 24 months | 391 | 0.8 (0.2, 1.4) | 382 | 0.3 (-0.4, 0.9) | 0.53 (-0.15, 1.20) | 0.10 |
| Physically active, % (95% CI) ^e^ |  |  |  |  |  |  |
| 12 months | 372 | 79.6 (68.3, 87.7) | 407 | 57.9 (51.9, 63.7) | 2.84 (1.49, 5.41) |  |
| 24 months | 391 | 75.8 (63.4, 85) | 382 | 47.5 (41.4, 53.8) | 3.45 (1.82, 6.56) | **<0.01** |
| BMI, mean (95% CI), kg/m^2^ |  |  |  |  |  |  |
| 12 months | 370 | 26.6 (25.3, 28.0) | 407 | 26.7 (25.4, 27.9) | -0.03 (-1.83, 1.78) |  |
| 24 months | 384 | 26.6 (25.3, 27.9) | 382 | 26.5 (25.3, 27.7) | 0.10 (-1.70, 1.91) | 0.91 |
| Waist circumference, mean (95% CI), cm |  |  |  |  |  |  |
| 12 months | 371 | 93.0 (90.6, 95.3) | 407 | 94.8 (92.0, 97.7) | -1.86 (-5.57, 1.85) |  |
| 24 months | 379 | 93.7 (91.3, 96.1) | 381 | 93.5 (90.6, 96.4) | 0.22 (-3.51, 3.95) | 0.91 |
| Current smoker, % (95% CI) |  |  |  |  |  |  |
| 12 months | 373 | 6.9 (3.4, 13.4) | 407 | 5.8 (3.2, 10.5) | 1.20 (0.45, 3.17) |  |
| 24 months | 392 | 6.2 (3.1, 12.3) | 382 | 6.2 (3.4, 11.0) | 1.01 (0.38, 2.69) | 0.98 |
| Fruits and vegetables intake (per week), mean (95% CI) ^f^ |  |  |  |  |  |  |
| 12 months | 373 | 6.5 (5.7, 7.3) | 407 | 7.2 (6.2, 8.1) | -0.67 (-1.93, 0.58) |  |
| 24 months | 392 | 6.0 (5.2, 6.8) | 382 | 6.7 (5.8, 7.6) | -0.70 (-1.89, 0.50) | 0.25 |
| Both fruits and vegetables intake at least once a week, % (95% CI) ^g^ |  |  |  |  |  |  |
| 12 months | 372 | 34.6 (27.2, 42.9) | 407 | 36.5 (28.9, 44.7) | 0.92 (0.57, 1.51) |  |
| 24 months | 392 | 30.8 (23.9, 38.8) | 381 | 34.3 (26.9, 42.5) | 0.85 (0.52, 1.40) | 0.53 |
| Sodium intake, mean (95% CI), g/day ^h^ |  |  |  |  |  |  |
| 12 months | - | - | - | - | - |  |
| 24 months | 339 | 4.1 (3.8, 4.3) | 246 | 4.0 (3.8, 4.3) | 0.04 (-0.35, 0.42) | 0.86 |
| Secondary clinical measures and outcomes |  |  |  |  |  |  |
| HDL cholesterol, mean (95% CI), mmol/L |  |  |  |  |  |  |
| 12 months | 332 | 1.4 (1.3, 1.5) | 354 | 1.4 (1.4, 1.5) | -0.05 (-0.16, 0.07) |  |
| 24 months | 376 | 1.4 (1.3, 1.5) | 369 | 1.4 (1.3, 1.5) | -0.02 (-0.13, 0.09) | 0.76 |
| LDL cholesterol, mean (95% CI), mmol/L |  |  |  |  |  |  |
| 12 months | 332 | 2.5 (2.4, 2.6) | 351 | 2.4 (2.3, 2.5) | 0.06 (-0.08, 0.21) |  |
| 24 months | 376 | 2.4 (2.3, 2.5) | 367 | 2.5 (2.4, 2.6) | -0.05 (-0.20, 0.10) | 0.52 |
| Total cholesterol, mean (95% CI), mmol/L |  |  |  |  |  |  |
| 12 months | 332 | 4.0 (3.7, 4.3) | 354 | 4.1 (4.0, 4.3) | -0.11 (-0.44, 0.23) |  |
| 24 months | 376 | 4.1 (3.8, 4.4) | 369 | 4.2 (4.0, 4.3) | -0.07 (-0.41, 0.27) | 0.69 |
| Triglycerides, mean (95% CI), mmol/L |  |  |  |  |  |  |
| 12 months | 332 | 1.5 (1.3, 1.6) | 354 | 1.4 (1.3, 1.5) | 0.03 (-0.14, 0.2) |  |
| 24 months | 376 | 1.5 (1.3, 1.6) | 369 | 1.4 (1.3, 1.5) | 0.03 (-0.15, 0.2) | 0.75 |
| Glycated Hemoglobin, mean (SD), % ^i^ |  |  |  |  |  |  |
| 12 months | 96 | 7.1 (6.8, 7.4) | 117 | 7.1 (6.8, 7.3) | 0.05 (-0.35, 0.45) |  |
| 24 months | 113 | 7.2 (6.8, 7.5) | 124 | 7.1 (6.9, 7.3) | 0.07 (-0.32, 0.46) | 0.73 |
| Fasting blood glucose, mean (95% CI), mmol/L |  |  |  |  |  |  |
| 12 months | 326 | 6.0 (5.7, 6.4) | 342 | 6.1 (5.8, 6.3) | -0.04 (-0.48, 0.40) |  |
| 24 months | 370 | 6.6 (6.0, 7.3) | 361 | 6.2 (5.5, 6.8) | 0.47 (-0.42, 1.36) | 0.30 |
| Estimated CKD-EPI GFR, mean (95% CI), ml/min/1.73m^2^ |  |  |  |  |  |  |
| 12 months | 320 | 80.0 (75.2, 84.8) | 352 | 78.9 (76.9, 81.0) | 1.09 (-4.16, 6.34) |  |
| 24 months | 377 | 78.3 (73.5, 83.2) | 368 | 77.8 (75.7, 79.9) | 0.53 (-4.77, 5.84) | 0.84 |
| Total antihypertensive therapeutic intensity score (TIS) score, mean (95% CI) ^j^ |  |  |  |  |  |  |
| 12 months | 373 | 1.1 (1.0, 1.2) | 407 | 0.9 (0.8, 1.0) | 0.16 (0.04, 0.29) |  |
| 24 months | 393 | 1.0 (0.9, 1.1) | 382 | 0.9 (0.8, 1.0) | 0.14 (0.01, 0.27) | **0.03** |
| 10- year ICVD risk score %, mean (95% CI) ^k^ |  |  |  |  |  |  |
| 12 months | 328 | 9.7 (7.3, 12.1) | 345 | 13 (10.5, 15.5) | -3.33 (-6.81, 0.15) |  |
| 24 months | 349 | 9.8 (7.4, 12.2) | 321 | 14 (11.5, 16.5) | -4.26 (-7.75, -0.77) | **0.02** |
| Incident diabetes, % (95% CI) ^l^ |  |  |  |  |  |  |
| 12 months | 447 | 1.8 (0.9, 3.5) | 469 | 1.3 (0.6, 2.9) | 1.41 (0.48, 4.15) |  |
| 24 months | 447 | 3.4 (2.0, 5.5) | 469 | 3.0 (1.7, 5.1) | 1.13 (0.53, 2.43) | 0.75 |
| **Participant reported health status** |  |  |  |  |  |  |
| Health related quality of life (HRQL) assessed by EQ-5D-5L VAS, mean (95% CI) ^m^ |  |  |  |  |  |  |
| 12 months | 370 | 78.7 (76.3, 81.1) | 407 | 76.4 (71.2, 81.6) | 2.34 (-3.36, 8.03) |  |
| 24 months | 390 | 80.1 (77.7, 82.6) | 382 | 73.9 (68.7, 79.0) | 6.28 (0.56, 11.99) | **0.03** |
| EQ-5D-5L utility index, mean (95% CI) ^n^ |  |  |  |  |  |  |
| 12 months | 373 | 0.93 (0.92, 0.94) | 407 | 0.94 (0.92, 0.96) | 0.0 (-0.02, 0.02) |  |
| 24 months | 392 | 0.94 (0.94, 0.95) | 382 | 0.94 (0.92, 0.96) | 0.0 (-0.02, 0.02) | 0.99 |
| **Process measures** |  |  |  |  |  |  |
| Number of antihypertensive medications, mean (95% CI) |  |  |  |  |  |  |
| 12 months | 373 | 1.91 (1.8, 2.01) | 407 | 1.74 (1.61, 1.87) | 0.17 (0.0, 0.33) |  |
| 24 months | 393 | 1.90 (1.79, 2.01) | 382 | 1.72 (1.59, 1.85) | 0.18 (0.01, 0.36) | **0.04** |
| Change from baseline in number of antihypertensive medications, mean (95% CI) |  |  |  |  |  |  |
| 12 months | 373 | 0.24 (0.13, 0.36) | 407 | 0.06 (0.02, 0.11) | 0.18 (0.05, 0.31) |  |
| 24 months | 393 | 0.24 (0.11, 0.36) | 382 | 0.04 (-0.02, 0.11) | 0.2 (0.06, 0.34) | **0.01** |
| SPC hypertensive medication, % (95% CI) |  |  |  |  |  |  |
| 12 months | 371 | 25.5 (17.7, 35.2) | 398 | 2.3 (1.0, 5.2) | 14.76 (5.53, 39.41) |  |
| 24 months | 384 | 23.8 (16.5, 33.2) | 376 | 2.1 (0.9, 5.1) | 14.27 (5.23, 38.97) | **<0.01** |
| Class of antihypertensive medications |  |  |  |  |  |  |
| Calcium Channel Blockers |  |  |  |  |  |  |
| 12 months | 373 | 58.2 (52.0, 64.1) | 407 | 62.3 (56.2, 68.1) | 0.84 (0.59, 1.20) |  |
| 24 months | 393 | 58.9 (52.8, 64.6) | 382 | 61.0 (54.7, 66.9) | 0.92 (0.64, 1.31) | 0.62 |
| RAAS inhibitors |  |  |  |  |  |  |
| 12 months | 373 | 66.9 (59.9, 73.3) | 407 | 58.8 (50.3, 66.9) | 1.42 (0.89, 2.24) |  |
| 24 months | 393 | 67.4 (60.5, 73.6) | 382 | 60.0 (51.4, 68) | 1.38 (0.87, 2.19) | 0.17 |
| Beta Blockers |  |  |  |  |  |  |
| 12 months | 373 | 33.8 (28.9, 39.1) | 407 | 37.8 (30.4, 45.7) | 0.84 (0.57, 1.26) |  |
| 24 months | 393 | 31.1 (26.4, 36.2) | 382 | 37.1 (29.8, 45.1) | 0.77 (0.51, 1.14) | 0.19 |
| HCTZ Diuretics |  |  |  |  |  |  |
| 12 months | 373 | 32.0 (23.9, 41.4) | 407 | 14.5 (11.4, 18.3) | 2.77 (1.7, 4.53) |  |
| 24 months | 393 | 30.9 (23.0, 40.1) | 382 | 15.2 (11.9, 19.1) | 2.50 (1.53, 4.09) | <0.01 |
| Cholesterol lowering medication, % (95% CI) |  |  |  |  |  |  |
| 12 months | 373 | 74.1 (68.6, 79) | 407 | 74.4 (69.1, 79.0) | 0.99 (0.68, 1.44) |  |
| 24 months | 393 | 75.9 (70.6, 80.6) | 382 | 75.5 (70.2, 80.1) | 1.03 (0.70, 1.50) | 0.89 |
| Number of oral antidiabetic medications, mean (95% CI) ^i^ |  |  |  |  |  |  |
| 12 months | 138 | 1.58 (1.28, 1.87) | 176 | 1.42 (1.25, 1.59) | 0.16 (-0.18, 0.50) |  |
| 24 months | 122 | 1.60 (1.31, 1.90) | 141 | 1.42 (1.25, 1.59) | 0.18 (-0.16, 0.52) | 0.29 |

Abbreviations: ACR, albumin to creatinine ratio; BMI, body mass index; BP, blood pressure; CI, confidence interval; CKD-EPI, Chronic Kidney Disease Epidemiology Collaboration; CVD, cardiovascular diseases; DBP, diastolic BP; FRS, Framingham Risk Score; GFR, glomerular filtration rate; HDL, high density lipoprotein; ICVD, integrated cardiovascular diseases, ITT, intention to treat; LDL, low density lipoprotein; MCI, Multicomponent Intervention; OR, Odds Ratio; SBP, systolic BP; SD, standard deviation; VAS, visual analogue scale.

^a^ Preliminary analysis results based on intention-to-treat principle.

^b^ Adjusted means difference between MCI and Usual Care for continuous outcomes and Odds ratio—MCI vs Usual care—for binary outcomes using repeated measures analysis.

^c^ The model accounts for mean baseline SBP for each participant, as a covariate in the model.

^d^ Physical activity was assessed using the International Physical Activity Questionnaire (IPAQ) score. The IPAQ Group, 2005 Guidelines for data processing and analysis of the international physical activity questionnaire (IPAQ) - short and long forms.

^e^ Physically active was defined as participants with ‘minimally/moderately active’ and ‘highly active’.

^f^ Frequency of fruit and vegetables consumption per week was summed up get the total frequency per week.

^g^ Frequency of participants consuming both fruits and vegetables at least once per week

^h^ The sodium intake is calculated using urine spot creatinine. 14 outlying values of sodium intake > 12 g/day (5 MCI, 9 Usual Care) were capped to 12.

^i^ The analysis was performed on participants with diabetes at baseline by American Diabetes Association (ADA) criteria. 314 participants were diabetic, 138 in MCI and 176 in Usual Care.

^j^ The outlying values of total TIS score at the top are capped at 90’th percentile. Levy PD, Willock RJ, Burla M, Brody A, Mahn J, Marinica A, Nasser SA, Flack JM. Total antihypertensive therapeutic intensity score and its relationship to blood pressure reduction. *Journal of the American Society of Hypertension : JASH* 2016; **10:** 906-916.

^k^ A 10-year integrated cardiovascular disease (ICVD) risk score for coronary events and ischemic stroke was computed using the equation developed for the Chinese adults. High 10-year ICVD risk was defined as > 20% of the 10-year ICVD risk score. Wu Y et al, 2006 Estimation of 10-Year Risk of Fatal and Nonfatal Ischemic Cardiovascular Diseases in Chinese Adults. *Circulation*. 2006; 114:2217-2225.

^l^ Incident diabetes was defined as use of hypoglycemic agents or fasting blood glucose ≥ 126 mg/dL during 2-year follow-up for participants without prevalent diabetes at baseline.

^m^ Health status was reported on a scale of 0 (worst imaginable health) to 100 (best imaginable health) using EQ-5D-5L, where higher values indicate better health.

^n^ EQ-5D-5L utility index was calculated using the Indonesian value set. It ranges from -0.865 (worst state) to 1 (full health), where higher values indicate higher health utility.

## Table D. Adverse and Serious Adverse Events by Intervention Group

| **Adverse event** | **Multicomponent Intervention**  **(N=447)** | **Usual Care**  **(N=469)** | ***P*-value ^a^** |
| --- | --- | --- | --- |
| Adverse event | 41 (9.2) | 39 (8.3) | 0.65 |
| Serious adverse event ^b^ | 58 (13.0) | 62 (13.2) | 0.91 |
| Deaths (all-cause) | 2 (0.5) | 6 (1.3) | 0.29 |
| **SAE ^c^** | 11 (2.5) | 13 (2.8) | 0.77 |
| Cardiovascular death | 0 (0) | 2 (0.4) | 0.50 |
| Coronary heart disease/MI/Acute coronary syndrome/IHD | 5 (1.1) | 2 (0.4) | 0.28 |
| Stroke/TIA | 6 (1.3) | 5 (1.1) | 0.70 |
| Heart failure | 0 (0) | 2 (0.4) | 0.50 |
| PVD/Gangrene | 0 (0) | 2 (0.4) | 0.50 |
| Peripheral edema | 0 (0) | 1 (0.2) | >.99 |
| **Hospitalization from AE potentially associated with intervention^d^** | 2 (0.5) | 6 (1.3) | 0.29 |
| Hypotension | 1 (0.2) | 1 (0.2) | >.99 |
| Dizziness/light-headedness | 1 (0.2) | 1 (0.2) | >.99 |
| Injuries/Falls | 0 (0) | 4 (0.9) | 0.12 |
| **AE potentially associated with intervention^e^** | 13 (2.9) | 24 (5.1) | 0.09 |
| Hypotension | 0 (0) | 1 (0.2) | >.99 |
| Dizziness/light-headedness | 4 (0.9) | 4 (0.9) | >.99 |
| Injuries/Falls | 2 (0.5) | 13 (2.8) | **0.01** |
| Bradycardia | 1 (0.2) | 0 (0) | 0.49 |
| Cough after initiating antihypertensive | 4 (0.9) | 1 (0.2) | 0.21 |
| Peripheral edema | 0 (0) | 4 (0.9) | 0.12 |
| Musculoskeletal pain | 2 (0.5) | 3 (0.6) | >.99 |
| **Other AEs with specific condition of interest** |  |  |  |
| Serum creatinine ^f^ | 3 (0.7) | 0 (0) | 0.12 |
| **AE requiring a visit to doctor or ED potentially associated with intervention ^f^** | 6 (1.3) | 13 (2.8) | 0.13 |

Abbreviations: AE, adverse event; ED, emergency department; IHD, ischemic heart disease; MCI, multicomponent intervention; MI, myocardial infarction; PVD, peripheral vascular diseases; TIA, transient ischemic attack.

^a^ Chi-square or Fisher’s Exact test was used to compare safety outcomes between randomized groups, as indicated

^b^ Serious adverse events (SAE) defined as adverse events falling into the following categories: 1) death; 2) life threatening event; 3) events resulting in permanent disability; 4) hospitalization; and 5) prolongation of hospital stay

^c^ Cardiovascular death, hospitalization for cardiovascular diseases due to acute coronary syndrome, elective revascularization, myocardial infarction, stroke, heart failure, peripheral vascular disease (PVD), and peripheral edema. One participant from usual care had reported two events 1) PVD 2) coronary heart disease. So the sum of participants reporting these events is 13 and not 14.

^d^ Hypotension, dizziness/ light-headedness, injury/fall

^e^ Hypotension, dizziness/light-headedness, injuries/falls, bradycardia, cough after initiating antihypertensive, peripheral edema, musculoskeletal pain

^f^ Serum creatinine > 20% of normal, but did not require any visit to doctor or emergency department

^g^ Hypotension, dizziness/light-headedness, injuries/falls, bradycardia, cough after initiating antihypertensive, peripheral edema, musculoskeletal pain, and associated with lab abnormalities as serum creatinine (>20% of normal), serum sodium (< 130 or > 150 mmol/L), serum potassium (< 3 or > 5.5 mmol/L)

## Table E. Intraclass correlation coefficient (ICC) of outcomes measured

| **Variable** | **ICC** |
| --- | --- |
| Systolic BP, mmHg | 0.009 |
| Systolic BP change from baseline | 0.022 |
| Diastolic BP, mmHg | 0.039 |
| ACR, mg/mmol | 0.007 |
| Log ACR | 0.005 |
| FRS, % | 0.017 |
| Estimated CKD-EPI GFR, ml/min/1.73m^2^ | 0.021 |
| 10- year ICVD risk score ^a^ | 0.036 |
| BMI, kg/m^2^ | 0.054 |
| Waist circumference, cm | 0.049 |
| Physical activity (MET/min per week) ^b^ | 0.053 |
| Physical activity (log MET/min per week) | 0.034 |
| Vegetables and fruits per week ^c^ | 0.032 |
| EQ-5D-5L VAS | 0.085 |
| EQ-5D-5L utility index ^d^ | 0.020 |
| HLI | 0.068 |
| Sodium intake ^e^ | 0.009 |
| Total cholesterol, mmol/L | 0.023 |
| HDL cholesterol, mmol/L | 0.034 |
| LDL cholesterol, mmol/L | 0.015 |
| Triglycerides, mmol/L | 0.026 |
| Glycated Hb | 0.026 |
| FBS, mmol/L | 0.008 |
| Total antihypertensive TIS score ^f^ | 0.021 |

Abbreviations: ACR, albumin to creatinine ratio; BMI, body mass index; BP, blood pressure; CKD-EPI, Chronic Kidney Disease Epidemiology Collaboration; CVD, cardiovascular disease; EQ-5D-5L, EuroQol 5-Dimension 5-Level questionnaire; FBS, fasting blood sugar; FRS, Framingham risk score; GFR, glomerular filtration rate; HDL, high density lipoprotein; LDL, low density lipoprotein; TIS, Total antihypertensive therapeutic intensity score; VAS, visual analogue scale

^a^ A 10-year integrated cardiovascular disease (ICVD) risk score for coronary events and ischemic stroke was computed using the equation developed for the Chinese adults. Wu Y et al, 2006 Estimation of 10-Year Risk of Fatal and Nonfatal Ischemic Cardiovascular Diseases in Chinese Adults. Circulation. 2006; 114:2217-2225.

^b^ Physical activity was assessed using the International Physical Activity Questionnaire (IPAQ) score. The IPAQ Group, 2005 Guidelines for data processing and analysis of the international physical activity questionnaire (IPAQ) - short and long forms.

^c^ Frequency of fruit and vegetables consumption per week was summed up get the total frequency per week.

^d^ EQ-5D-5L utility index was calculated using the Indonesian value set. It ranges from -0.865 (worst state) to 1 (full health), where higher values indicate higher health utility.

^e^ The sodium intake is calculated using urine spot creatinine.

^f^ Levy PD, Willock RJ, Burla M, Brody A, Mahn J, Marinica A, Nasser SA, Flack JM. Total antihypertensive therapeutic intensity score and its relationship to blood pressure reduction. Journal of the American Society of Hypertension: JASH 2016; 10: 906-916.

## Table F. Raw means (SD) of primary and BP-Related Key Secondary Outcomes

|  | **n** | **Total (N=916, 8 clinics)** | **n** | **Multicomponent Intervention (N=447, 4 clinics)** | **n** | **Usual Care (N=469, 4 clinics)** |
| --- | --- | --- | --- | --- | --- | --- |
| Systolic BP, Mean (SD), mmHg | 766 | 137.0 (15.8) | 384 | 135.3 (16.0) | 382 | 138.7 (15.5) |
| Diastolic BP, Mean (SD), mmHg | 766 | 81.6 (10.5) | 384 | 82.2 (9.94) | 382 | 81.0 (11.1) |
| BP controlled to conventional goal (SBP <140 mmHg and DBP <90 mmHg), n (%) | 766 | 429 (56.0) | 384 | 229 (59.6) | 382 | 200 (52.4) |
| High FRS 10-year CVD risk, n (%) ^a^ | 728 | 380 (52.2) | 363 | 170 (46.8) | 365 | 210 (57.5) |
| Ln Urine ACR, mean (%), mg/mmol | 721 | 1.41 (1.2) | 366 | 1.31 (1.0) | 355 | 1.52 (1.4) |
| Number of anti-hypertensive medications per day, mean (SD) | 775 | 1.83 (2.0) | 393 | 1.90 (2.0) | 382 | 1.75 (2.0) |

Means shown in this table are based on crude data at 24 months and not adjusted for clutering effects.

Abbreviations: ACR, albumin to creatinine ratio; BP, blood pressure; CVD, cardiovascular disease; FRS, Framingham risk score; Ln, Natural logarithm.

^a^ High 10-year Framingham risk score (FRS) CVD risk score was defined as >20% risk of CVD at 10 years.

## Table G. Program Delivery Costs in Singapore Dollar (SGD)

| **Activity** | **No. of sessions** | **Cost per session** | **Singapore**  **(actual)**  **(SGD)** |
| --- | --- | --- | --- |
| **A. ADMIN & OVERSIGHT** | | | **13,459.23** |
| Labor for listing and rostering subjects for telephone calls, filling up checklists, documenting in EMR, coordinating transfer of checklists, and tracking monthly task completion, and Site PI allocated for meetings/discussion | NA | NA | 10,459.23 |
| Labor for site PI/physician to assess progress of intervention delivery and discussions with nurses | 12 | 250.00 | 3,000.00 |
|  |  |  |  |
| **B. TRAINING** |  |  | **47,484.43** |
| **Nurses motivational conversation (MC) training** | |  | **27,884.43** |
| *Initial training-1 day session* | 3 | 3,600.00 | 10,800.00 |
| *Refresher training- half day session* | 5 | 1,880.00 | 9,400.00 |
| *In kind cost* | 8 | 50.00 | 400.00 |
| *Opportunity cost for trainees* |  |  | 7,284.43 |
|  |  |  |  |
| **Physician training** |  |  | **19,600.00** |
| *Initial training (two 1-hour sessions per clinic)* | 8 | 300.00 | 2,400.00 |
| *Refresher training (one 1-hour session per clinic)* | 8 | 300.00 | 2,400.00 |
| *In kind cost* | 16 | 150.00 | 2,400.00 |
| *Printing training manuals & algorithm displays* | 16 | 37.50 | 600.00 |
| *Training team travel costs (Duke to SHP clinic)* | 16 | 50.00 | 800.00 |
| *Opportunity cost for trainees* |  |  | 11,000.00 |
|  |  |  |  |
| **C. IMPLEMENTATION OF MOTIVATIONAL CONVERSATION** | | | **85,140.77** |
| **Labor** | | | **80,140.77** |
| Mean minutes of face to face in-clinic MC per high risk hypertensive participant (only once in year 1, includes documenting in EMR) | | | 30 |
| Number of high risk hypertensive participants | | | 200 |
| Manhours for face to face in-clinic MC | | | 100 |
| Mean minutes of telephone based follow up MC per hypertensive participant (over 24 months, including missed call 3 attempts, filling checklists, documenting in EMR) | | | 5 |
| Number of hypertensive participants | | | 400 |
| Number of telephone based follow up MC per hypertensive participant | | | 10 |
| Manhours for telephone based follow up MC | | | 333.33 |
| Total manhours | | | 433.33 |
| Costs associated with face to face in-clinic MC based on manhours | | | 18,494.02 |
| Costs associated with telephone based follow up MC based on manhours | | | 61,646.75 |
| **Materials and supplies** | | | **5,000.00** |
| Telephone based line charges follow up total cost for 2 years | | | 4,000.00 |
| Materials and supplies | | | 1,000.00 |
|  |  |  |  |
| **D. GENERAL PRACTITIONER VISIT COSTS** |  |  | **7,107.00** |
| **Visit costs** |  |  | **7,107.00** |
|  |  |  |  |
|  | **Cost per unit** | **No of units** |  |
| **E. SINGLE PILL COMBINATION DRUG (HYZAAR)** | | | **15,770.00** |
| Medication subsidy (50%) for Hyzaar standard dose per tablet | 0.2 | 50,434 | 7,581.02 |
| Medication subsidy (50%) for Hyzaar forte dose per tablet | 0.3 | 18,944 | 4,271.40 |
|  | | | |
| **F. LABORATORY TESTS RELATED TO SINGLE PILL COMBINATION DRUG (HYZAAR)** | | | **7,905.00** |
| Safety lab test for individuals who started on Hyzaar | 51 | 155 | 7,905.00 |
|  |  |  |  |
| **G. EXCESS COSTS FOR OTHER ANTI-HYPERTENSIVES, STATINS** | | | **7,765.96** |
| Excess costs for other anti-hypertensives, statins |  |  | 7,765.96 |
|  |  |  |  |
|  |  |  |  |
| **GRAND TOTAL** | **A+B+C+D+E+F+G** |  | **184,632.39** |
|  |  |  |  |
| **Cost of intervention per year per participant** |  |  | **230.79** |
|  |  |  |  |

# Section S9. References:

1. Snow V, Aronson MD, Hornbake ER, Mottur-Pilson C, Weiss KB, Clinical Efficacy Assessment Subcommittee of the American College of P. Lipid control in the management of type 2 diabetes mellitus: a clinical practice guideline from the American College of Physicians. Ann Intern Med 2004;140:644-9.
2. Chen YJ, Li LJ, Tang WL, et al. First-line drugs inhibiting the renin angiotensin system versus other first-line antihypertensive drug classes for hypertension. Cochrane Database Syst Rev 2018;11:CD008170.
3. Mancia G, Fagard R, Narkiewicz K, et al. 2013 ESH/ESC Guidelines for the management of arterial hypertension: the Task Force for the management of arterial hypertension of the European Society of Hypertension (ESH) and of the European Society of Cardiology (ESC). J Hypertens 2013;31:1281-357.
4. Chobanian AV, Bakris GL, Black HR, et al. The Seventh Report of the Joint National Committee on Prevention, Detection, Evaluation, and Treatment of High Blood Pressure: the JNC 7 report. JAMA 2003;289:2560-72.
5. Graham JW, Olchowski AE and Gilreath TD. How many imputations are really needed? Some practical clarifications of multiple imputation theory. Prev Sci. 2007;8:206-13.
6. Payscale. Average senior charge nurse (RN) salary in Singapore. 2020. <https://www.payscale.com/research/SG/Job=Senior_Charge_Nurse_(RN)/Salary> (accessed April 28 2021).
7. Payscale. Average general practitioner salary in Singapore. 2020. <https://www.payscale.com/research/SG/Job=General_Practitioner/Salary> (accessed April 28 2021).
8. Payscale. Average registered nurse (RN) salary in Singapore. 2021. [https://www.payscale.com/research/SG/Job=Registered_Nurse_(RN)/Salary](https://www.payscale.com/research/SG/Job=Registered_Nurse_(RN)/Salary%20) (accessed April 28 2021).
9. Polyclinic Singhealth. Patient care charges & payment. 2021. <https://polyclinic.singhealth.com.sg/patient-care/charges-payment> (accessed April 7 2021).
