# Supplementary material for: Integration of a multicomponent intervention for hypertension into primary healthcare services in Singapore—A cluster randomized controlled trial
Source: PLoS Med. 2022 Jun 13;19(6):e1004026. doi: 10.1371/journal.pmed.1004026 (PMC9239484; doi:10.1371/journal.pmed.1004026)
Supplement: Attachment — Submitted filename: Reviewer comments_PlosMed.docx [file pmed.1004026.s003.docx]

**Response to Editors’ and Reviewers’ Comments:**

Comments are in bold and responses in italics.

**Comments from the Academic Editor:**

**This is a well conducted, pragmatic study of a multifaceted intervention. I think the effect size is in keeping with pragmatic health care interventions of these nature and so I am not that concerned by reviewer 4 comments on that particular issue. The longer follow-up is certainly a value add as the authors point out most studies are of much shorter duration.**

*Thank you for appreciating the high rigor of our trial, its noveltly of longer term follow-up than previously reported studies, and that the observed effect size is clinically meaningful especially for interventions in real world settings.*

**I do agree with reviewer 2 that the major gap in the paper is more information on implementation fidelity. There is absolutely no information on numbers of people reached by each of the intervention components including medication use and adherence rates and whether fidelity changed over time which could help explain why effects emerged in year 2 (assuming much of the year 1 reduction was regression to the mean). It really is a bit of a black box trying to work out the causal path to effectiveness and this does not help readers with generating new knowledge or replicating the study in other settings (as recommended by the authors).**

*Thank you for these comments. Several details about intervention and implementation fidelity were presented in Supplement Table S2, and we have added more information now. Although the trial was not designed to assess the causal pathways of the benefit of the intervention, our exploratory analyses show that at 24 months, relative to control participants, participants in the intervention group had 1) greater numbers of antihypertensive medications, 2) greater therapeutic intensity of antihypertensive medications (accounting for both number and dose of medications), 3) greater single-pill combination (SPC) antihypertensive medications, and, 4) were, possibly, more physically active (based on higher proportion physically active in the intervention group at 24 months albeit change in log MET score was not statistically different between groups) (see table below and Table 2 and Supplement Table S3).*

**Additional analyses of secondary outcomes ^a^**

|  | **n** | **Multicomponent**  **Intervention**  **(N=447, 4 clinics)** | **n** | **Usual care**  **(N=469, 4 clinics)** | **Adjusted Means Difference**  **/OR ^b^ (95% CI)** | ***P*-value** |
| --- | --- | --- | --- | --- | --- | --- |
|  | | | | | | |
| Number of antihypertensive medications, mean (95% CI) | | | | | | |
| 12 months | 373 | 1.91 (1.80, 2.01) | 407 | 1.74 (1.61, 1.87) | 0.17 (0.00, 0.33) |  |
| 24 months | 393 | 1.90 (1.79, 2.01) | 382 | 1.72 (1.59, 1.85) | 0.18 (0.01, 0.36) | **0.04** |
| Change from baseline in number of antihypertensive medications, mean (95% CI) | | | | | | |
| 12 months | 373 | 0.24 (0.13, 0.36) | 407 | 0.06 (0.02, 0.11) | 0.18 (0.05, 0.31) |  |
| 24 months | 393 | 0.24 (0.11, 0.36) | 382 | 0.04 (-0.02, 0.11) | 0.20 (0.06, 0.34) | **0.01** |
| Total antihypertensive therapeutic intensity score (TIS) score, mean (95% CI) ^i^ | | | | |  |  |
| 12 months | 373 | 1.1 (1.0, 1.2) | 407 | 0.9 (0.8, 1.0) | 0.16 (0.04, 0.29) |  |
| 24 months | 393 | 1.0 (0.9, 1.1) | 382 | 0.9 (0.8, 1.0) | 0.14 (0.01, 0.27) | **0.03** |
| Prescribed SPC for hypertension, % (95% CI) ^d^ | | |  |  |  |  |
| 12 months | 371 | 25.5 (17.7, 35.2) | 398 | 2.30 (1.00, 5.20) | 14.76 (5.53, 39.41) |  |
| 24 months | 384 | 23.8 (16.5, 33.2) | 376 | 2.10 (0.90, 5.10) | 14.27 (5.23, 38.97) | **<0.01** |
| Physical activity (change in log MET/min per week from baseline), mean (95% CI) ^d^ | | | | |  |  |
| 12 months | 372 | 1.0 (0.4, 1.6) | 407 | 0.7 (0.1, 1.3) | 0.27 (-0.41, 0.95) |  |
| 24 months | 391 | 0.8 (0.2, 1.4) | 382 | 0.3 (-0.4, 0.9) | 0.53 (-0.15, 1.20) | 0.10 |
| Physically active, % (95% CI) ^e^ |  |  |  |  |  |  |
| 12 months | 372 | 79.6 (68.3, 87.7) | 407 | 57.9 (51.9, 63.7) | 2.84 (1.49, 5.41) |  |
| 24 months | 391 | 75.8 (63.4, 85) | 382 | 47.5 (41.4, 53.8) | 3.45 (1.82, 6.56) | **<0.01** |
|  |  |  |  |  |  |  |

*We did not have valid measures of adherence, however self-reported use of antihypertensive medications was >95% at baseline in both groups and remained as such.*

*Nevertheless, taken together, our exploratory analysis suggests that the benefit of intervention is likely to be mediated primarily through increased number of antihypertensive medications, and possibly complemented by some lifestyle changes.*

**The cost analysis is cursory. It only assesses costs of the intervention and does not include costs of care utilisation, medications, diagnostic tests etc. The EQ5D-5L VA scores only are reported which shows a difference but the mean scores are not reported. It is difficult to see how this intervention might generate a quality of life gain in such a short period of time with just a small reduction in BP. That said a cost per QALY estimate could be relatively easily generated from the data available to determine an incremental cost effectiveness ratio and I suggest this be included in the paper to help us understand this in more detail.**

*As mentioned in the paper, the cost analysis is primarily from the health planners' perspectives. We have now included the cost of all antihypertensive and lipid-lowering medications in the cost analysis and clarified the other items (care utilization and diagnostic tests) as well.*

*The mean baseline EQ5D-5L VAS scores at baseline were reported in the Supplement Tables. We now report them in the main paper (Table 1) as well.*

*EQ5D-5L VAS scores are based on self-perceived health status. Our study was not designed to assess how the intervention might improve quality of life. Motivational conversation by trained nurses which focused on self-care and empathy could have potentially led to improvement in perceived quality of life.*

*The within-trial incremental cost per QALY for intervention is likely to underestimate the cost-utility of intervention as these estimates do not account for the benefits of hypertension reduction is likely to accrue over many years. However, if any of the benefits are short-lived, which is likely the case for behavioral improvements, then the CE ratio would be overestimated. Therefore, we have not discussed the cost per QALY estimates in the paper. A detailed incremental cost-effectiveness analysis taking a lifetime perspective will be the subject of another paper (stated on page 16 line 340 in the revised manuscript). However, we do provide updated detailed costs and QALY estimates, separately, in this paper.*

**A few other issue of note:
1. I also was quite perplexed by the large rise in albuminuria in the usual care group. This is quite a spurious finding and not at all in keeping with the effect sizes observed in the other outcome variables. This warrants greater attention.**

*We agree that the observed effect size on ACR is large.*

*Given the non-normality of albuminunuria (urine albumin to creatinine ratio (ACR)), we more appropriately analysed ln ACR in the revised manuscript. The difference remains significant. Exponentiating the log-difference at 24 months (-0.22) gives an MCI/UC ratio of 0.83 (95% CI: 0.664, 0.980) that translates to an estimated 19.7% (33.6%, 2%) reduction in urine ACR. The effect of the MCI remains statistically significant, however the true magnitude of the effect size remains uncertain, pending additional study, especially since ACR was a secondary outcome. We have explained the plausibility of potential benefit in response to reviewer 4.*

*Nevertheless, in the revised paper (page 32, line 605), we have added under limitations that “Fifth, the `benefit of the intervention on albuminuria reduction could be a chance association, pending further studies for confirmation.”*

|  | **n** | **Multicomponent**  **Intervention**  **(N=447, 4 clinics)** | **n** | **Usual care**  **(N=469, 4 clinics)** | **Adjusted Means Difference**  **/OR ^b^ (95% CI)** | ***P*-value** |
| --- | --- | --- | --- | --- | --- | --- |
|  | | | | | | |
| Ln urine ACR, mean (95% CI) | | | | | | |
| Baseline ^c^ | 442 | 1.35 (1.18, 1.52) | 467 | 1.43 (1.28, 1.58) | -0.08 (-0.26, 0.1) |  |
| 24 months | 366 | 1.32 (1.16, 1.48) | 355 | 1.53 (1.39, 1.68) | -0.22 (-0.41, -0.02) | **0.03** |

**2. I was also surprised that other cardimetabolic parameters such as lipids and blood glucose were not included. This information was collected and lipids are stated as a secondary outcome measure on p.11 but it is not reported. I have not checked the protocol and SAP but worth checking if all outcomes measures are reported as per protocol.**

*We have now reported changes in blood glucose and glycated hemoglobin (details below in response to reviewers' comments and Supplement Table S3). Information on lipids (total, LDL, HDL cholesterol) was already included in the Supplement. (Supplement Table S3).*

**3. The authors state total mortality was lower for intervention (0.5%) compared to usual 437 care (1.3%) (p=0.29) - this should be amended as there was no statistically significant difference in mortality.**

*The statement has been amended to “total mortality was not different between the intervention (0.5%) vs. usual 437 care (1.3%) (p=0.29)” page 26, line 477.*

**Requests from the editors:

Please structure your abstract using the PLOS Medicine headings (Background, Methods and Findings, Conclusions).**
 **Please report your abstract according to CONSORT for abstracts, following the PLOS Medicine abstract structure (Background, Methods and Findings, Conclusions)** <https://ddec1-0-en-ctp.trendmicro.com:443/wis/clicktime/v1/query?url=http%3a%2f%2fwww.consort%2dstatement.org%2fextensions%3fContentWidgetId%3d562&umid=8c679298-2feb-479d-b7ba-061d75f22951&auth=3eee6d57317a38631073652579c10dc620ca2b41-f4d753be2a36a42dce9db8848f128a1616e58035>

**Abstract Background:**

**Provide the context of why the study is important. The final sentence should clearly state the study question.

Abstract Methods and Findings:**

**Please state that there was no masking in the trial.
Please define both the intervention and control states.
Please provide the number of participants lost to follow up in each group.
Please indicates the dates during which study enrollment and follow up occurred.
Please include the important dependent variables that are adjusted for in the analyses.
Please include a summary of adverse events.
Please ensure relevant p values are reported.**

*Thank you. These abstract has been modified accordingly.*

**In the last sentence of the Abstract Methods and Findings section, please describe the main limitation(s) of the study's methodology.**

*The main limitation is now stated at the end of the Abstract “The main limitation was no masking in the trial”.*

**The Data Availability Statement (DAS) requires revision. If the data are not freely available, please describe briefly the ethical, legal, or contractual restriction that prevents you from sharing it. Please also include an appropriate contact (web or email address) for inquiries (this cannot be a study author).**

*DAS has been revised accordingly.*

**Author Summary
Thank you for including an Author Summary. Please update as follows:
* Relocate such that Author Summary is placed after Abstract and before Introduction.
* Combine bullets one and two in a single-sentence bullet.
* In the final bullet under ‘Why was this study done?’, please replace ‘were’ with ‘are’ and relocate the comma after ‘mixed’ to come after ‘(majority <6 months)’.
* Bullets under the ‘What did the researchers do and find?’ can be slightly more succinct (aim for 3 single sentence bullet points, if possible).
* Please quantify the main findings (i.e. lowering of blood pressure and improvement in BP control relative to usual care) and consider stating ‘was not associated with any safety concerns’ rather than ‘was safe’ (same comment applies at Line 471).**

*The Author Summary has been revised accordingly and now placed after the abstract.*  **Please remove funding details from the first page (this information is captured in your Financial Disclosure statement in the submission form).**

*Funding details have been removed from the first page.*
 **Citations should be in square brackets and preceding punctuation.**

Citations have been reformated accordingly.

**Thanks for completing the CONSORT checklist. Please cite your checklist in the Methods (‘This trial is reported according to the Consolidated Standards of Reporting Trials (S1 Checklist)’ at line 224.**

**Thank you. The CONSORT checklist has been in Methods section as per instructions stated above.**

**Please update the checklist to use section and paragraph numbers, rather than page numbers.**

*The checklist has been updated accordingly.*

**Please define what is meant by dropouts/loss to follow up.**

*Lost to follow-up/dropouts were participants on whom primary outcome (BP) could not be ascertained at 24 months post-baseline. This has now been clarified in the revised paper.*

*The reasons for loss to follow-up are presented in the CONSORT figure 1.*

**Please present the safety data for the study including numbers of specific events and whether or not adverse events are thought to be related to treatment.**

## *The safety data is presented in* *Supplement Table S4. Adverse and Serious Adverse Events by Intervention Group with adverse events potentially associated with the intervention.*

**Please indicates the dates during which study enrollment and follow up occurred.**

The enrolment in the study started in Jan 2017 and ended in May 2018. The follow-ups started in Dec 2018 and ended in Sep 2020. This is now mentioned in the abstract (pg 3, line 50) and results (pg 17, line 346, and line 349) in the revised manuscript.

**Line 217: Please cite your protocol as an item within the Supporting Information (see our guidance here** <https://ddec1-0-en-ctp.trendmicro.com:443/wis/clicktime/v1/query?url=https%3a%2f%2fjournals.plos.org%2fplosmedicine%2fs%2fsupporting%2dinformation&umid=8c679298-2feb-479d-b7ba-061d75f22951&auth=3eee6d57317a38631073652579c10dc620ca2b41-81500a2627cb2610a670f57abfa53585e3250bc6)>.

*The protocol has been added as an item within the Supporting Information.*

**Line 210: Outcomes could be described more clearly (they are easier to determine in the protocol/on clinicaltrials.gov).**

The outcomes in the paper are more detailed and follow the description in the Statistical Analysis Plan (SAP). However, we have simplified them in the revised paper.

 **Line 271: Please cite the SAP you have included in your Supporting Information.**

*SAP has been added to the supporting information.*
 **Line 281: Should Sensitivity Analyses have their own subsection?**

*Yes, we believe it is better to report Sensitivity Analysis as a separate subsection. We followed the same format in our previous publication in NEJM* [*https://www.nejm.org/doi/10.1056/NEJMoa1911965*](https://www.nejm.org/doi/10.1056/NEJMoa1911965)*?*

**Given that randomisation has been performed, please remove p values from Table 1 and Table S1.**

*We have removed p values from Table 1 and Table S1.*

**Please clarify the difference between Table 1 and Table S1.**

Table S1 includes several additional variables and granular information on baseline characteristics that are not included in Table 1. 

**Please ensure consistency when reporting p values in terms of decimal places (e.g. Table 3).**

*Thank you. We have made reporting of p values consistent throughout the paper including in Table 3.*

**Line 433: Should this not be P>0.05 for all given threshold for significance?**

*Thanks yes rephrased.*  **Line 437: Why is this reported as a difference when P=0.29?**

*Thanks- this has been amended to no difference.*

**Supplementary sections S1-4 are not cited in the text as far as I can see?**

*Thank you. We have cited all sections as supporting documents before the Acknowlegements.*

**Please remove funding details from the Acknowledgements.**

*Funding details have been removed from the Acknowlegements.*

**The Data Availability Statement can be removed from the end of the main text – please ensure all relevant information is captured in the relevant question on the Submission Form.**
 *The Data Availability Statement has been removed from the main text.*

Comments from the reviewers:

Reviewer #1:

**Prof. Tazeen H Jafar and his colleague conducted the trail to assess the effectiveness of a multicomponent intervention on blood pressure control compared to usual care, and the findings showed a significant lowering of SBP level and a higher control rate of BP in management group than that in usual group. Several comments:**

1. **How about the comparability of the Clinics between two groups?  What`s the proportion of education degree of the physicians, how many years did they work, how about the social economic level, etc.**

There were four clinics in each group. All clinics belonged to the same administrative health cluster and were regulated by the Ministry of Health. All physicians in polyclinics have a minimum of an MBBS diploma. We did not collect information on the duration of service, however, it is expected to be comparable between clinics. The hiring of physicians is done centrally using uniform criteria with no distinction by socioeconomic level. The socio-economic level of the patients was also comparable at baseline as reflected by the type of housing, employment rate, and education status (table 1 and Supplement table S1)

2. **Although four trainings (beginning, 3m, 12m, 24m) for doctors, three times (beginning, 12m, 24m) for nurses, were done in intervention group, except the last one, only 3 times for doctors, 2 times for nurses, if there were the other effect beside the "intervention". Subsidies on drug?**

*In terms of the “multicomponent intervention”, it is correct that doctors were trained on updated management of hypertension, nurses were trained on motivational counselling and telephone-based follow-ups, subsidies single-pill combination antihypertensive drug, and the trained doctors and nurses deliver these interventions to the patients.*

3. **According to the design, there was no power to test the effect on the secondary outcomes, why added many items?**

It is correct that we did not have the power to study multiple secondary outcomes, especially changes in lifestyle and hard CVD outcomes, as that would require a much larger study. However, we were interested in exploring potentially large effects on process outcomes (such as changes in medications and lifestyle) that would provide clues regarding the pathways of the benefit, as well as vascular outcomes (such as albuminuria) and additional indicators (quality of life) to support other dimensions of the benefit of the multicomponent intervention. We pre-specified the outcomes in the statistical analysis plan.

**Reviewer #2:**

**This was a cluster-based randomized program which compared multicomponent intervention vs usual care for control of BP in Singapore setting. In a nutshell, 8 clinics participated, 4 were intervention clinics and 4 were usual care clinics. The intervention included training physicians to stratify risk, 50% subsidy for fixed dose combination (FDC) of ARB and thiazide, motivational interview by nurses and regular telephone calls. A clinical research coordinator also performed evaluation at month 0, 12 and 24 in both groups. The intervention group was supposed to be seen by doctors/nurses at 3-4 monthly intervals. Over 400 patients were recruited in each group and the endpoint was SBP reduction at 24 months along with Framingham 10-year risk score and ACR. There was also mention of EQ5D evaluation and cost effectiveness analysis. About 80% of patients returned at month 24 for evaluation. The study was influenced by the COVID-19 outbreak.**

**The analysis was very technical using multiple statistical methods to compare clinic-based rather than individual patient-level data with adjustment for clusters and time trends. Analyses were also performed using imputed data with multiple modelling as well as using data of patients who returned at month 24 in a per protocol analysis. The conclusion was the intervention group achieved lower SBP by 3 mmHg and had an 1.5 odds ratio of achieving a BP<140/90 with 40% risk reduction of 10-year CVD risk score and reduction in ACR compared with usual care.

There is now consensus that the challenges in control of NCD lie in implementation which needs support from healthcare planners and payors in addition to providers and patients. Multicomponent interventions are also well accepted although its implementation depends on context which needs to take into consideration the usual care model for scalability and sustainability. It is here that the paper lacks granularity as to what the usual care model is. Do people in Singapore have a regular family doctor or do they shop around for doctors?**

**For WHO defined essential lab tests (e.g. lipid, A1c, RFT) and medications (statins, ACEi, metformin, SU etc), do they have to co-pay or are they fully covered?**

**Is there a ceiling for government coverage and do patients have to pay every time out of pocket to see their doctor or a nurse?**

*Thank you for appreciating our cluster-randomized trial of a multicomponent intervention in a clinic setting, its rigorous and robust statistical analysis, and our clinically meaningful findings.*

*We have now provided more clarification on healthcare in Singapore in the revised manuscript (page 9, lines 164 to 173). The vast majority of adults with chronic disease in Singapore seek care at the polyclinics. At the government polyclinics, patients are assigned to a pool of physicians who, along with the nurses and other healthcare staff, provide subsidized care and medications. Patients may also choose to be seen by a family physician in the polyclinic at a significantly higher (2 to 3 times) cost.*

*The essential laboratory tests and medications per the WHO list also have a co-pay, albeit almost 80% of the cost is covered by the government.*

*Singapore does not have a ceiling for government coverage, however patients have to co-pay a nominal amount (15 to 25%) depending upon age, citizenship status, and income group.* [*https://polyclinic.singhealth.com.sg/patient-care/charges-payment*](https://polyclinic.singhealth.com.sg/patient-care/charges-payment)

**Do patients get medications from the clinic or pharmacy and can they self purchase medications without a prescription? Are there huge differences amongst these clinics in terms of staff to patient ratio, care pathways and payment structure? All these factors may influence the quality of care in the intervention and usual care groups.**

*Medications for chronic conditions are dispensed strictly on clinicians’ prescriptions from the pharmacies often situated within a clinic facility. Medications are not dispensed without a prescription. This is clarified on page 9 (lines 164 to 173). in the revised manuscript. Polyclinics in Singapore are regulated by the Ministry of Health. There are no major differences among the polyclinics in terms of staff to patient ratio, care pathways, and payment structure, or how patients get medications from the clinics.*

**In its current format, it is challenging to visualize the implementation procedures and patient/provider adherence as well as pre and end of study clinical profiles in both groups. Apart from BP and some demographic data, there was no information on eGFR, lipids and glycaemic indexes, some of which were needed to compute the 10-year CVD risk score. There was mention of EQ5D but not reported in the paper.**

*Thank you. Kindly note that information on eGFR, lipids (LDL, HDL, total cholesterol), and incident diabetes was provided in Supplement Table S3.*

*The EQ5D was measured as both visual analog score (VAS) and utility index was reported as means (SD) in Supplement Table 1 (baseline) and follow-up in Supplement Table S3 (12 and 24 months). We have now reported the EQ5D baseline means in the main paper Table 1 as well.*

*We have now added information on fasting blood glucose and glycated hemoglobin (measured in patients with diabetes only). (Supplement Table S3) The latter was measured only among individuals with diabetes. The findings were not different between groups.*

|  | **n** | **Multicomponent**  **Intervention**  **( 4 clinics)** | | **n** | | **Usual care**  **(N=4 clinics)** | | **Adjusted Means Difference**  **/OR ^b^ (95% CI)** | | | ***P*-value** |
| --- | --- | --- | --- | --- | --- | --- | --- | --- | --- | --- | --- |
| Glycated Hemoglobin, mean (SD), % | | | | | | | | | |  |  |
| 12 months | 96 | 7.1 (6.8, 7.4) | 117 | | 7.1 (6.8, 7.3) | | 0.05 (-0.35, 0.45) | |  |  |  |
| 24 months | 113 | 7.2 (6.8, 7.5) | 124 | | 7.1 (6.9, 7.3) | | 0.07 (-0.32, 0.46) | | 0.73 |  |  |
| Fasting blood glucose, mean (95% CI), mmol/L | | | | | | | | | |  |  |
| 12 months | 326 | 6.0 (5.7, 6.4) | 342 | | 6.1 (5.8, 6.3) | | -0.04 (-0.48, 0.40) | |  |  |  |
| 24 months | 370 | 6.6 (6.0, 7.3) | 361 | | 6.2 (5.5, 6.8) | | 0.47 (-0.42, 1.36) | | 0.30 |  |  |

**The cost-effectiveness analysis was very minimal and it is not clear what was the average number of man-hour involved (doctors/nurses), number of face to face visits and telephone consultations per patient during this 2-year program in the intervention group. What was the no of clinic visits in the usual care group? These information should have been captured by the project coordinator, indeed, what information was collected during these important evaluation visits at 0, 12 and 24 month.**

As mentioned on page 27, we have presented the incremental cost of the intervention, which is relevant for health planners for implementation and scaling up the intervention. We have now provided more details on the components included in the cost analysis (Supplement Section S7-A2). We have also listed the number of face-to-face patient visits and manhours during telephone follow-ups under Supplement Table S2.

During the 2 years of the trial, participants in the intervention group visited the clinics 8.5 times in the intervention clinics compared to 8.3 times in usual care, albeit not statistically different (p>0.05).

Those in the intervention group received one face-to-face session for motivational conversation. (Supplement Table S2)

## The number of telephone consultations, during the trial, was 7.2 (95% CI, 7.0, 7.4) as provided in Supplement Table S2.

A detailed incremental cost-effectiveness analysis taking a lifetime perspective will be the subject of another paper. We have stated this in the revised paper on pg 16, line 340.

## **The authors proposed that the model is applicable to many countries. If this is their message, then they have to be very specific on the implementation details. How many nurses and doctors were involved in each of these participating clinics? What is the provider:patient ratio? What were the drop out and adherence rates and why did people drop out? Why did patients do not join the program?**

*Thank you for seeking these clarifications. According to the World Bank, the physician and nurse to population ratios in Singapore are 2.4 and 6.2 per 1000 population, respectively, which are lower than in most high-income countries. In the polyclinics, physicians have a high caseload with up to 40-50 patients per physician daily.*

*These details are now provided in the revised paper (pg 9, lines 164 to 173, and pg 30, line 569)*

*The trial flow chart including pre-screened, those eligible and enrolled, and the reasons for drop-out are presented in CONSORT figure 1. The reasons for not joining the program due to lack of consent were not recorded on ethical grounds. The two-year dropout rates in the intervention and control groups were 14.1% and 18.6%, respectively. Implementation fidelity was high as shown in Supplement Table S2. All participants in the intervention group received care from a trained physician and a session of face-to-face motivational conversation. They received on average 7.2 telephone follow-ups over 2 years by trained nurses. Of the eligible participants at high risk of CVD, 63.7% received SPC antihypertensive medication, almost all of whom received the associated subsidy.*

**From a clinical perspective, were there any changes in other medications for lipids, BP and glucose?**

*As explained above, the prescriptions for antihypertensive medications increased more for patients in the intervention group than usual care. These results are shown in Table 1 and Table 2 in the main paper.*

*However, there was no difference in the proportion of participants prescribed lipid-lowering drugs, probably because the use of these agents was already high at baseline (74% of participants) as shown in Supplement Table S 1.*

*There was no difference in the mean number of oral anti-diabetes medications at 24 months (Supplement Table S1). This analysis was restricted to patients with diabetes at baseline.*

|  | **n** | **Multicomponent**  **Intervention**  **(4 clinics)** | **n** | **Usual care**  **( 4 clinics)** | **Adjusted Means Difference**  **(95% CI)** | ***P*-value** |
| --- | --- | --- | --- | --- | --- | --- |
| Number of oral antidiabetic medications, mean (95% CI) | | | | | |  |
| 12 months | 138 | 1.58 (1.28, 1.87) | 176 | 1.42 (1.25, 1.59) | 0.16 (-0.18, 0.5) |  |
| 24 months | 122 | 1.60 (1.31, 1.9) | 141 | 1.42 (1.25, 1.59) | 0.18 (-0.16, 0.52) | 0.29 |

**What were the changes in these risk factors that contribute to the 40% reduction in 10-year CVD risk score?**

*The FRS uses gender-specific scores based on age, systolic BP, cholesterol, HDL cholesterol, smoking status, and diabetes status. Although we have not performed a formal mediation analysis to examine the relative contibutors to the change in FRS scores, it is likely to be a combination of several factors especially BP lowering in older men and potential improvements in cholesterol, even though the study lacked power to detect significance changes in the individual risk factors.*

**Was there any difference in new cases of diabetes between the 2 groups?**

*As shown in Supplement Table S3, no difference in new cases of diabetes (incident diabetes) was observed between the two groups.*

**Since patients have to pay out of pocket for 50% of the FDC, how many patients agreed to the switch and persisted with the new medications?**

*As per protocol, FDC (or single pill combination (SPC)) was offered to patients at high risk for CVD. A total of 201 (44.9%) out of 447 pateints qualified, of whom 63.7% agreed to switch or start SPC (Supplement Table S3). The vast majority of patients persisted and did not discountinue during the course of the trial after switching.*

**On this point, did patients have access to a list of free medications for BP control?**

*As explained above, medications are not provided free of cost. Patients have access to list of subsidized medications through the physician and the pharmacy.*

**At baseline, the patients were already on multiple drugs and what was the pattern of drug usage between the 2 groups at baseline and end of 24 months?**

*As shown in Supplement Table S1, at baseline 62% were on calcium channel blockers, 53% on RAAS blockers, 38% on beta-blockers, and 13% on HCTZ diuretics. The use at 12 and 24 months are shown below (Supplement Table S3).*

*The pattern of antihypertensive mediations changed with a relative increase in SPC antihypertensive medications (including RAAS blockers and HCTZ diuretics), and HCTZ diuretics in the intervention group compared to usual care at 24 months.*

| **Class of antihypertensive medications** | **n** | **Multicomponent**  **Intervention**  **(N=447, 4 clinics)** | **n** | **Usual care**  **(N=469, 4 clinics)** | **Adjusted Means Difference**  **/OR ^b^ (95% CI)** | ***P*-value** |  |
| --- | --- | --- | --- | --- | --- | --- | --- |
| SPC antihypertensive medications, % (95% CI) ^d^ | | | | | | | |
| 12 months | 371 | 25.5 (17.7, 35.2) | 398 | 2.3 (1.0, 5.2) | 14.76 (5.53, 39.41) | |  |
| 24 months | 384 | 23.8 (16.5, 33.2) | 376 | 2.1 (0.9, 5.1) | 14.27 (5.23, 38.97) | | **<0.01** |
|  | | | | | | | |
| Calcium Channel Blockers |  |  |  |  |  | |  |
| 12 months | 373 | 58.2 (52.0, 64.1) | 407 | 62.3 (56.2, 68.1) | 0.84 (0.59, 1.20) | |  |
| 24 months | 393 | 58.9 (52.8, 64.6) | 382 | 61.0 (54.7, 66.9) | 0.92 (0.64, 1.31) | | 0.62 |
| RAAS inhibitors |  |  |  |  |  | |  |
| 12 months | 373 | 66.9 (59.9, 73.3) | 407 | 58.8 (50.3, 66.9) | 1.42 (0.89, 2.24) | |  |
| 24 months | 393 | 67.4 (60.5, 73.6) | 382 | 60.0 (51.4, 68) | 1.38 (0.87, 2.19) | | 0.17 |
| Beta Blockers |  |  |  |  |  | |  |
| 12 months | 373 | 33.8 (28.9, 39.1) | 407 | 37.8 (30.4, 45.7) | 0.84 (0.57, 1.26) | |  |
| 24 months | 393 | 31.1 (26.4, 36.2) | 382 | 37.1 (29.8, 45.1) | 0.77 (0.51, 1.14) | | 0.19 |
| HCTZ Diuretics |  |  |  |  |  | |  |
| 12 months | 373 | 32.0 (23.9, 41.4) | 407 | 14.5 (11.4, 18.3) | 2.77 (1.7, 4.53) | |  |
| 24 months | 393 | 30.9 (23.0, 40.1) | 382 | 15.2 (11.9, 19.1) | 2.50 (1.53, 4.09) | | **<0.01** |

*And our results also show (Table 3 and see response above) that the number of anti-hypertensive medications increased by 0.2 per patient in the intervention group relative to the usual care.*

**In the intervention group, were the patients informed of their BP goals and were they taught to self monitor BP? Indeed having a coordinator to relay these 2 messages every 6 months or so might go a long way while allowing the doctors and patients make their own choices in term of FU visits, drug regimens and lab tests. Indeed, this may be the reason why both groups had dropped their BP significantly with the yearly review by the project coordinator.**

*In the intervention group, formal training on home BP monitoring was not done per se. However, standardized messages regarding the importance of BP goals, and self-care were delivered, and home BP monitoring was encouraged by the trained nurses.*

*It is also possible that just the 6- monthly outcomes assessment by the research coordinators (outcomes assessors) with questions on lifestyle and BP could have influenced behavioral changes in patients and contributed to some of the BP-lowering observed in both the intervention and usual care groups. We have already mentioned in the limitations on pg 31 that “Moreover, usual care participants also experienced a decline in BP, which was most likely due to behavior modification in response to BP screening by the clinical research coordinators. These factors would contribute to the underestimation of the full benefit of the intervention. Moreover, regression to the mean could account for some reduction in BP, although it would apply equally to both arms”*

**The authors should list the names of all 8 participating clinics and their PIs in the acknowledgement.**

*Thank you for the suggestion. The names of all 8 participating clinics are mentioned in the acknowledgements. Their PIs were part of the inverstigators’ teams and are contributing co-authors or collaborators and mentioned as such in the paper and Supplement.*


**Reviewer #3: Alex McConnachie, Statistical Review

Jafar et al present the results of a cluster randomised trial of a multicomponent intervention in primary care to improve blood pressure control. This review considers the statistical aspects of the paper.

Overall, this is an excellent study, very well reported. The statistics are generally very good, and my comments are therefore relatively minor.**

*Thank you so much for appreciating our work.*

**One difficulty with cluster randomised trials is always the timing of randomisation at the cluster level and recruitment of individual patients. My impression from the paper is that practices were randomised first, and then the patients were recruited. What is not clear is whether the patients were aware of the allocation of their practice at the time of giving consent to be included in the study, or when they provided their baseline data. Also, how long was it between randomisation of the practices and recruitment/baseline? What was the timing of the intervention - had any training taken place before the patients had completed their baseline assessments? Were the follow-ups conducted 12- and 24-months after each patient's baseline assessment, or relative to the randomisation of the practice? Line 228 refers to the 2-year follow-up post-randomisation, but was it actually 2 years post-baseline?**

*Yes, this is correct. Randomization was done about 6 months before commencing recruitment. The patients were aware of the group assignments at recruitment. The consent forms stated the same. Motivational conversation (MC) training for nurses was provided 4-5 months before the commencement of recruitment. The physicians' training in the intervention group was done about 4 weeks before commencing recruitment for the baseline assessments. However, none of the participants received MC or telephone follow-ups from the trained nurses before the baseline assessment. Likewise, none of the participants recruited in the trial were managed by the trained physicians until after the baseline assessment.*

*The 12- and 24-months follow-ups were timed after each patient's baseline assessment (i.e post-baseline), and not since randomization (post-randomization). We have now clarified this in the revised manuscript on pg 8 and pg 9, lines 151 to 153.*

**Line 231 refers to blood pressure <140/90 as an outcome, though there was an earlier reference (line 159) to <130/80 as a target for some patients. Was this lower target used for relevant patients in defining this outcome?**

*As stated on page13, line 268, of the manuscript, the pre-specified primary outcome for the trial was “*SBP based on a mean of last 2 of 3 measurements at the 2-year post-baseline final follow-up”.

*Pre-specified key BP-related secondary outcomes included the percentage of participants with BP control (SBP <140 mmHg and DBP <90 mmHg ie <140/90). These results are shown in table 1 and table 2 in the main paper.*

*In addition, we conducted several additional analyses using three different thresholds for defining BP control including that used in the treatment algorithm for patients at high risk of CVD (<130/80), and our findings were consistent as shown in Supplement Table S3.*

**The sample size calculation appears correct, but it is not possible to replicate exactly without knowing what loss to follow-up was assumed.**

*A loss to follow up of 20% was assumed. This is provided in the statistical analysis plan cited in the main paper [18] and Supplement.*

**The repeated measures approach to the analysis is good, and is described very well. The sensitivity analyses are all very sensible, though the definition of "per protocol" is not clear - I did find it in the footnote to one of the tables, but it could perhaps be included in the main text. In sensitivity analysis 4, I assume the covariates were baseline measures?**

*Thank you again for appreciating our analysis. As suggested, we have added the definition of per-protocol analysis “Participants who were high CVD risk as per the checklist and did not receive single-pill combination (SPC) in the multi-component intervention (n=73) as per protocol, were excluded from this analysis” in the main paper on page 15, line 324.*

*We have clarified that sensitivity analysis 4 adjusted for clinically important variables that were measured at baseline. (page 16, line 326)*

**Some people would complain about reporting p-values for baseline differences, but I think it is a good thing with cluster randomised trials, and it is good that these comparisons take account of the clustering.**

*On suggestion of the editor, we have now removed p values from comparison of baseline characteristics.*
 **In Tables 2 and 3, we are given the mean and 95% CI, or percentage with 95% CI, in each randomised arm. This is not wrong, but for me, it does not show the distribution of the data in each group, since the CI depends on the sample size. Personally, I would rather see a summary of the data, i.e. mean and SD, or number and percentage, within each group.**

*The means presented in tables 2 and 3 are based on linear mixed model repeated measures analysis with treatment group, follow-up, treatment group×follow-up interaction as fixed effects, and random cluster (clinic) effects We have now added standard error (SE) of the means and proportions in the tables in addition to the 95% CI.*

**Reporting ICC statistics (line 377) is good, though it would be good if these could be reported for all outcomes, perhaps within the supplement somewhere**.

*We have now reported ICCs for all continuous outcomes in supplement table S6.*

**Finally, in Figure 1, the variance in cluster sizes in the two arms are reported as 21 and 13 "per cluster". I'm not sure that "per cluster" makes sense, since the units of variance is the square of the units of measurement. Would it be easier to report the SD of cluster sizes?**

*We have now reported SD of cluster size in Figure 1.*

**Reviewer #4:**

**Summary:
This study investigates the effects of multicomponent intervention for managing hypertension. In this cluster randomized controlled trial (RCT) within a primary care setting in Singapore, an intervention that integrates CVD risk assessment, subsidised single pill combination drugs, motivational conversations and telephone follow-up was compared to standard care in lowering blood pressure (BP). After a follow-up of up to two years, the investigators report a reduction of 3 mmHg systolic BP and better control of hypertension in the active arm compared to standard care.

Comments:
This trial is indeed interesting in that factors beyond that of the drug therapy itself is being considered and reflect effectiveness of an intervention that are relevant to practice settings. The inclusion of CVD risk assessment is an important strength, as treatment benefit tends to be higher in people with a higher background risk, at least in absolute terms. However, an important limitation is that it recruited participants with baseline BP levels of 140/90 mmHg or higher. I would have thought that CVD risk assessment would have benefited those at increased risk but their baseline BP may be slightly below this BP thresholds (e.g., hypertension treatment may be initiated in patients with diabetes whose BP are below this threshold as some clinical guidelines would suggest).

The achieved BP difference (of just over 3 mmHg systolic) seems very modest - clinically important at a group level, but likely less so at individual level. Because the aim of the intervention is to control hypertension or bring BP levels below the 140/90 mmHg threshold, which is the same for both treatment arms, it seems that the added benefit of all the components only amounted to 3 mmHg of effectiveness - a huge undertaking for a modest impact. Rather than focusing on the various components, would it have been better to focus on a component of the intervention that is most likely to achieved a greater reduction, perhaps by ensuring reaching an optimal target that is well below 140/90 mmHg? Very few (<6%) were at 'low-risk' of CVD at baseline, and the vast majority were either at medium or high risk. What would have been the impact if everyone was simply offered single pill combination drugs - it would have simplified further the intervention, and even if the costs might be slightly higher, the benefit in reducing BP perhaps might be freater?**

*We beg to differ with these comments.*

*As mentioned in the paper (abstract and results) “the adjusted mean systolic BP was significantly lower in the intervention group compared to usual care (-3.3 mmHg; 95% CI: -6.34, -0.32; p=0.03). The intervention led to higher BP control (odds ratio 1.51; 95% CI: 1.10, 2.09; p=0.01), and lower odds of high (>20%) 10-year cardiovascular risk score (OR 0.67; 95% CI: 0.47, 0.97; p=0.03).” These effects are very significant and clinically meaningful in terms of the projected benefit on cardiovascular morbidity and mortality.*

*Trials in adults with uncontrolled hypertension demonstrate a steep health improvement from BP reductions, where even a 1 mm Hg reduction in systolic BP decreased the risk of stroke by 5%. (Psaty BM, et al. JAMA 2003;289:2534-44). In over 1 million adults, a 2- mm Hg decline in systolic BP was associated with a 7% reduction in risk of coronary heart disease and mortality, and a 10% reduction in risk of stroke and related mortality. (Lacey B, et al. Lancet Glob Health 2018;6:e641-e9. Thus, as mentioned in the paper, 2- mm Hg lower systolic BP has been associated with a reduction of 7% to 10% in the risk of coronary heart disease, stroke, and related mortality.*

*Our analysis of the intervention effect on various subgroups did not show any significant interaction indicating that the intervention was uniformly effective in those with a very low risk of CVD. In fact, according to our study protocol, patients at low risk of CVD (50% of trial participants) did not qualify for SPC yet demonstrated BP reduction. Therefore, intervention components other than SPC are important in this subgroup.*

**The inclusion criteria included BP levels of ≥140/≥90 mmHg based on mean of last 2 of 3 measurements. Since a good proportion of participants were on antihypertensive medications at baseline, does it mean that either these patients had poor BP control and/or had higher baseline BP if they were not on medications?**

*That is correct, all eligibile patients has uncontrolled hypertension (as already stated in the abstract, methods, and discussion).*

**Making the most of BP measurements in reporting BP differences is indeed more appropriate than simply comparing BP changes from baseline. However, it is expected that BP measured a few months after randomisation tend not to reflect the full effects of the intervention since several adjustments/titration take place during this period, as well as regression to the mean/random variability in BP. I suggest that the authors consider a sensitivity analysis that exclude BP measured, say, in the first 3 or 6 months - it might be possible to demonstrate a bigger treatment effect than the 3 mmHg systolic BP being reported.**

*Thank you. As mentioned above the 3 mm Hg difference in systolic BP is consistent with the effect size from interventions in pragamatic settings.*

**The difference in the albumin:creatinine ratio seems very substantive which is not in keeping with differences in other parameters between treatment arms. Was this an expected finding? Is this a plausible result given the small difference in achieved BP reduction between the comparison groups?**

*Thank you for seeking the clarification. As explained in response to the editor’s comments, given the non-normality of ACR, we more appropriately analysed ln ACR in the revised manuscript. The difference remains significant.*

|  | **n** | **Multicomponent**  **Intervention**  **(N=447, 4 clinics)** | **n** | **Usual care**  **(N=469, 4 clinics)** | **Adjusted Means Difference**  **/OR ^b^ (95% CI)** | ***P*-value** |
| --- | --- | --- | --- | --- | --- | --- |
|  | | | | | | |
| Ln urine ACR, mean (95% CI) | | | | | | |
| Baseline ^c^ | 442 | 1.35 (1.18, 1.52) | 467 | 1.43 (1.28, 1.58) | -0.08 (-0.26, 0.1) |  |
| 24 months | 366 | 1.32 (1.16, 1.48) | 355 | 1.53 (1.39, 1.68) | -0.22 (-0.41, -0.02) | **0.03** |

*The benefit of intervention on ACR is likely to be mediated both by BP lowering and the use of blockers of the renin-angiotensin-aldosterone system such as losartan which are known to have an antiproteinuric effect that is in addition to their BP-lowering effect, especially when used together with diuretics. [56] [57]. These agents are used more frequently in patients in the intervention group than in usual care as a component of the SPC drug.*

*Nevertheless, we have added the following statement on pg 32, line 605, under limitations “Fifth, the `benefit of the intervention on albuminuria reduction could be a chance association, pending further studies for confirmation. However, blockers of the renin-angiotensin-aldosterone system (RAAS) such as losartan are known to have antiproteinuric effects that are in addition to their BP-lowering effect, especially when used together with diuretics. [56] [57]. RAAS blockers and diuretics were part of the SPC medications used more frequently in patients in the intervention group”.*

- [56]	Trujillo H, Caravaca-Fontan F, Caro J, Morales E, Praga M. The Forgotten Antiproteinuric Properties of Diuretics. Am J Nephrol. 2021;52(6):435-49. Epub 20210707. doi: 10.1159/000517020. PubMed PMID: 34233330.
- [57] .Eijkelkamp WB, Zhang Z, Remuzzi G, Parving HH, Cooper ME, Keane WF, et al. Albuminuria is a target for renoprotective therapy independent from blood pressure in patients with type 2 diabetic nephropathy: post hoc analysis from the Reduction of Endpoints in NIDDM with the Angiotensin II Antagonist Losartan (RENAAL) trial. J Am Soc Nephrol. 2007;18(5):1540-6. Epub 20070404. doi: 10.1681/ASN.2006050445. PubMed PMID: 17409317.
